# Influence of Magnetic Field Strength on Magnetic Resonance Imaging Radiomics Features in Brain Imaging, an *In Vitro* and *In Vivo* Study

**DOI:** 10.3389/fonc.2020.541663

**Published:** 2021-01-20

**Authors:** Samy Ammari, Stephanie Pitre-Champagnat, Laurent Dercle, Emilie Chouzenoux, Salma Moalla, Sylvain Reuze, Hugues Talbot, Tite Mokoyoko, Joya Hadchiti, Sebastien Diffetocq, Andreas Volk, Mickeal El Haik, Sara Lakiss, Corinne Balleyguier, Nathalie Lassau, Francois Bidault

**Affiliations:** ^1^ Department of Radiology, Gustave Roussy Cancer Campus, Université Paris-Saclay, Villejuif, France; ^2^ BioMaps (UMR1281), Université Paris-Saclay, CNRS, INSERM, CEA, Orsay and Gustave Roussy, Villejuif, France; ^3^ Immunology of Tumours and Immunotherapy INSERM U1015, Gustave Roussy Cancer Campus, Université Paris Saclay, Villejuif, France; ^4^ Radiology Department, Columbia University Medical Center, New York Presbyterian Hospital, New York, NY, United States; ^5^ Center for Visual Computing, CentraleSupelec, Inria, Université Paris-Saclay, Gif-sur-Yvette, France; ^6^ Department of Radiotherapy - Medical Physics, Gustave Roussy, Université ParisSaclay, Villejuif, France

**Keywords:** tissue features, heterogeneous phantom, homogeneous phantom, magnetic fields, texture, magnetic resonance imaging

## Abstract

**Background:**

The development and clinical adoption of quantitative imaging biomarkers (radiomics) has established the need for the identification of parameters altering radiomics reproducibility. The aim of this study was to assess the impact of magnetic field strength on magnetic resonance imaging (MRI) radiomics features in neuroradiology clinical practice.

**Methods:**

T1 3D SPGR sequence was acquired on two phantoms and 10 healthy volunteers with two clinical MR devices from the same manufacturer using two different magnetic fields (1.5 and 3T). Phantoms varied in terms of gadolinium concentrations and textural heterogeneity. 27 regions of interest were segmented (phantom: 21, volunteers: 6) using the LIFEX software. 34 features were analyzed.

**Results:**

In the phantom dataset, 10 (67%) out of 15 radiomics features were significantly different when measured at 1.5T or 3T (student’s t-test, p < 0.05). Gray levels resampling, and pixel size also influence part of texture features. These findings were validated in healthy volunteers.

**Conclusions:**

According to daily used protocols for clinical examinations, radiomic features extracted on 1.5T should not be used interchangeably with 3T when evaluating texture features. Such confounding factor should be adjusted when adapting the results of a study to a different platform, or when designing a multicentric trial.

## Highlights

- Radiomic features at 1.5T are not interchangeable with 3T when evaluating tumor texture
- Field strength should be taken into account in the interpretation of the texture indices
- Signal to noise ratio should be taken into account in the interpretation of the texture indices

## Introduction

Radiomics is a fast growing discipline, which is undergoing growing interest in computational medical imaging (1). This field of medical study aims at extracting a large amount of quantitative features from medical images using data-characterization algorithms. This research field is faced with multiple challenges (2). Radiomics is used in oncology to analyze features that are invisible to the naked eye and that may be associated with gene expression, tumor histology, treatment response and patient outcome (3).

MRI has several advantages and disadvantages for radiomics analysis (4–7). Among imaging modalities, it offers the best soft tissue contrast. Conversely, differences in MRI parameters (field strength, gradient characteristics), image acquisition protocols (8, 9), sequences, pixel size (10), and the signal-to-noise ratio (SNR) (11, 12) might impact radiomics features that are sensitive to image quality.

The impact of MRI acquisition and processing on radiomics reproducibility is scarcely reported. As in nuclear medicine, the validation of a biologically relevant and reproducible clinical biomarker based on radiomics implies standardization of protocols across several centers (13). For example, 3T compared with 1.5T MRI not only provides higher SNR, allows increased image resolution, and modifies relaxation times T1 and T2 but also induces some artifacts. Thus, there is a clear need to evaluate the influence of field strength and related settings like image resolution (pixel size, field of view [FOV], and matrix) on radiomic features.

Recent articles using radiomics as biomarkers consider that the major challenge is that grayscale MRI intensities, contrary to X-ray CT, are not standardized and are highly dependent on manufacturer, sequence type and acquisition parameters (14, 15).

To address this problem, authors focused on image pre-processing techniques that effectively minimize MR intensity inhomogeneity in a tissue region (16–18), spatial resampling (17–20) and brain arch extraction prior to image intensity normalisation (21, 22).

Although several studies have shown variability in texture analysis as a function of MRI acquisition parameters and gray level discretization steps, none of them have evaluated the combined impact of magnetic field strength, matrix size, pixel size, intensity normalization, and gray level discretization pre-processing methods on MRI radiomic feature values (9, 10, 23).

This study was designed to evaluate the impact of the field strength (1.5T vs. 3T) on radiomic features. Two clinical 1.5 and 3T MR devices from the same manufacturer were used to image the same phantoms, mimicking homogeneous and heterogeneous tissues. We also imaged healthy volunteers with the same sequences acquisition and image processing parameters.

It is believed that this step is crucial before proposing recommendations to standardize brain MRI pre-processing techniques, which is in turn essential for guaranteeing reliable radiomics-based models with big data and artificial intelligence.

## Material and Methods

This prospective study was approved by the local institution review board.

### Homogeneous Phantoms

**Figure 1:**
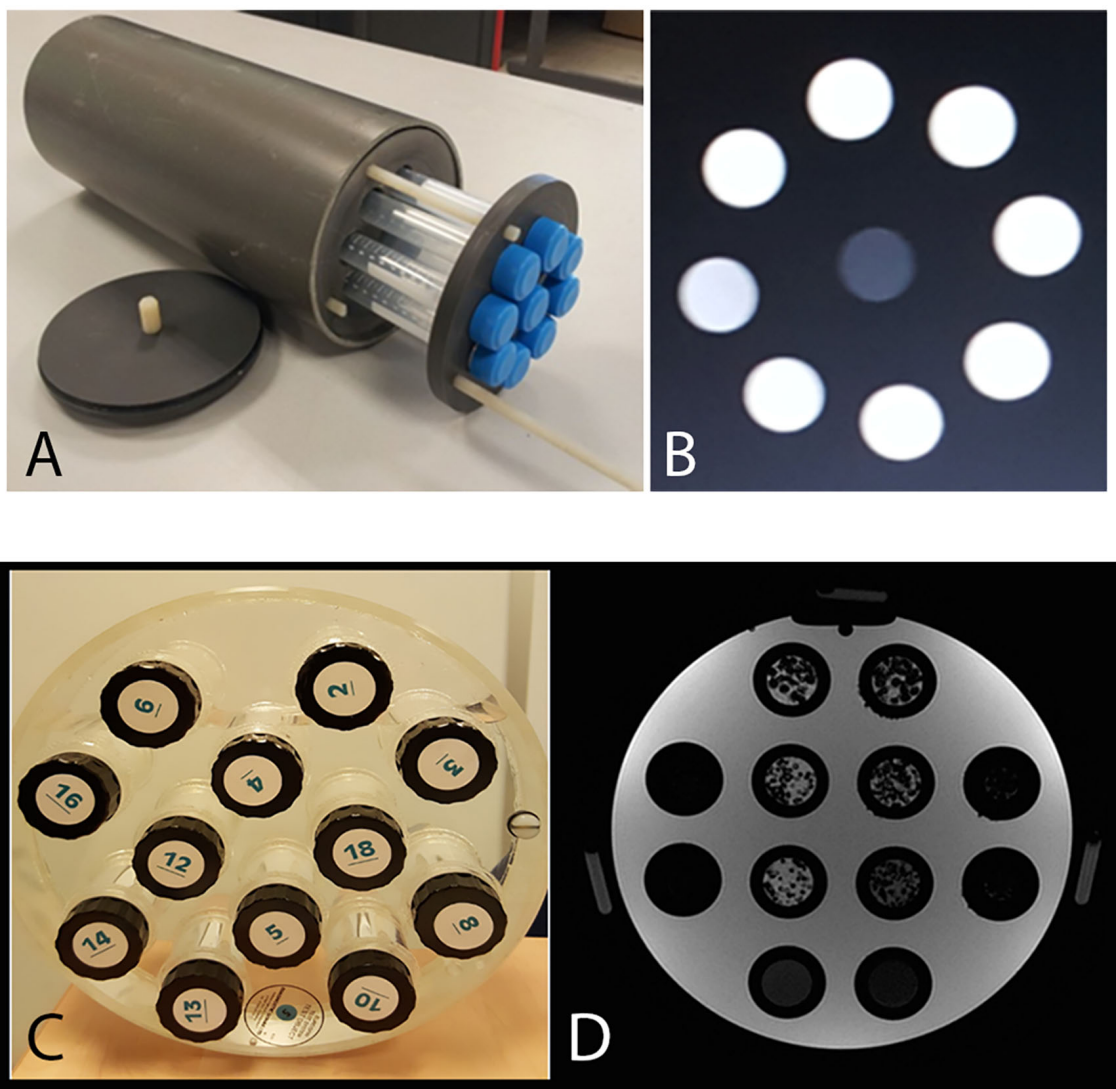
**(A)** Homogeneous phantoms: eight 30-ml tubes filled with demineralized water mixed with increasing gadolinium chelate concentration (Gadoteric Acid; Dotarem^®^, Guerbet): 0.25, 0.5, 0.75, 1, 1.25, 1.5, 1.75 and 2 mmol/l and a central demineralized water tube. **(B)** T1 weighted MRI image of the homogeneous phantom (256x256 matrix, 18 cm FOV). **(C, D)** T1 weighted MRI image of the heterogeneous phantom (256;256 matrix, 24 cm FOV): in the two central columns are six 30-ml tubes filled with polystyrene beads of different diameters (1, 2 and 3 mm) in an agarose gel solution (2%), either pure or mixed with 0.25 mmol/l gadolinium chelate, and two 30-ml tubes filled with agarose gel solution (2%) and two different concentration of gadolinium chelate.

Our homogeneous phantom was designed to mimic cerebrospinal fluid and opacified blood vessels. It was defined with different gadolinium concentrations: eight 30-ml tubes were filled with demineralized water mixed with increasing gadolinium chelate concentration (Gadoteric Acid; Dotarem^®^, Guerbet): 0.25, 0.5, 0.75, 1, 1.25, 1.5, 1.75, and 2 mmol/l (**Figures 1A, B**). Those eight homogeneous tubes were designated C1 to C8.

### Heterogeneous Phantoms

Our heterogeneous phantom, designed to mimic the brain white matter, was composed of agarose gel coating polystyrene beads. The proton density of agarose has similar characteristics and relaxation times to biological tissues (10). We defined six heterogeneous tubes and two homogeneous tubes. The 30-ml tubes were filled with polystyrene beads of different diameters (1, 2 and 3 mm) in an agarose gel solution (2%), either pure or mixed with 0.25 mmol/l Gadolinium chelate (**Figures 1C, D**) (9). The tubes were displayed in the tube slots of the Eurospin phantom (A Eurospin II-(TO5) phantom; Diagnostic Sonar) glass cylinder, filled with 1% copper sulfate (24). The eight tubes were designated T1 to T8.

### Healthy Volunteers

Clinical MRI sequences were performed in 10 healthy volunteers aged 21–26 years (six men, four women). Two MRI acquisitions were performed on each MRI device within a 40-min time interval.

### MRI Devices and Protocols

The influence of field strength was tested on two different MRI devices from the same manufacturer (General Electric): an Optima MR450w 1.5T superconducting magnet MRI installed in 2016 with a 70 cm tunnel, 32 channels, 50 cm FOV (Z axis), and gradients 40 mT SR 200 mT/m/s, and a Discovery MR750w 3T superconducting magnet MRI installed in 2012, with a 70 cm tunnel, 32 channels, 50 cm FOV (Z axis), and gradients 44 mT/m SR 200 T/m/s.

We used ‘head and neck’ coils with 32 channels with a 35 cm diameter adapted to the frequency of each MRI.

### Acquisition

MRI imaging acquired was based on a T1-weighted 3D rapid gradient echo sequence (3D SPGR). This sequence is used in clinical imaging for rapid volumetric imaging and can be acquired before or after contrast agent injection. The same sequences parameters (matrix, FOV, plane) were used for both MRIs. Parameters were chosen as recommended by the European Organization for Research and Treatment of Cancer (EORTC) to explore brain tumors: repeat time (TR) 6.1 ms; echo time (TE) inphase 1.2–2.1 ms; NEX1; thickness 1 mm in contiguous sections and bandwidth at 31 with 165 slices; 2–5-min acquisition depending on the FOV and matrices. The temperature was maintained between 19 and 21°C in each MRI during acquisition. For the healthy volunteers, the same 3DT1 SPGR sequence was used covering the whole encephalon.

### Acquisition Parameters: Field of View and Matrix Size

For the phantom study, five couples of matrices and FOV, determining various pixel size, were applied on both machines (**Table 1**). The couples were chosen to be suitable with clinical acquisition. In healthy volunteers only one acquisition per device was performed, the FOV was fixed at 24 cm with an image matrix of 256 × 256 on both machines.

**Table 1:** Matrices and FOVs studied at 1.5 and 3T with the corresponding pixel size.

MTX (pixels)	Field of view (cm)	Pixel size (mm)
256 x 256	24	0.938
256 x 256	18	0.703
256 x 256	12	0.468
256 x 128	24	0.937 x 1.875
128 x 128	24	1.875

### Texture Analysis

Segmentations and texture features calculations were performed with the freely available LIFEX software package (http://www.lifexsoft.org) (25). A 6 cm^3^ VOI was placed in each homogeneous and heterogeneous phantom’s tube. VOI position along Z axis of tubes was controlled according to the reference markers located on the phantom surface. For healthy volunteers, VOI were displayed in six bilateral areas: corpus callosum, central gray nuclei, and white matter of the centrum semiovale.

In each VOI, 38 texture features (indices) were extracted from first order and second-order statistics, arising from the analysis of the intensity histogram and the calculation of three texture matrices: the co-occurrence matrix (CM), the gray-level run length matrix (GRLM) and the gray-level zone length matrix (GZLM) (26). The texture analysis software needs to resample the 16-bit gray levels of MRI images in order to calculate these features. Voxel intensities were resampled in three different ways, using 256, 128 and 64 discrete values.

### Calculation of Signal-to-Noise Ratio

Mean SNR values were calculated on both MRIs by measuring the ratio between the mean signal intensity in each pure agarose sample and the standard deviation (SD) of the background noise selected in the frequency encoding direction. Calculation of SNR in healthy volunteers was also performed by measuring the mean signal intensity in each white substance and the SD of the background noise selected in the frequency encoding direction.

### Statistical Methods

Two-by-two correlation texture features were calculated. The statistical significant differences between 1.5 and. 3T were determined by the Student’s t-test using paired data. In the event of a lack of normal distribution (N < 30), results were also presented as boxplots to demonstrate the trend of texture values on 1.5T vs. 3T. The association between acquisition protocols (magnetic field, matrix, FOV), textural changes in the phantom (homogeneous vs. heterogeneous and polystyrene size), and imaging features, was calculated with Spearman’s rho correlation coefficients with P-values corrected for multiple tests. Statistical analyses were performed using SPSS v24.0. The datasets generated and analyzed are available from the corresponding author.

## Results

### Phantom Study

#### Influence of Software Resampling Step

**Figure 2:**
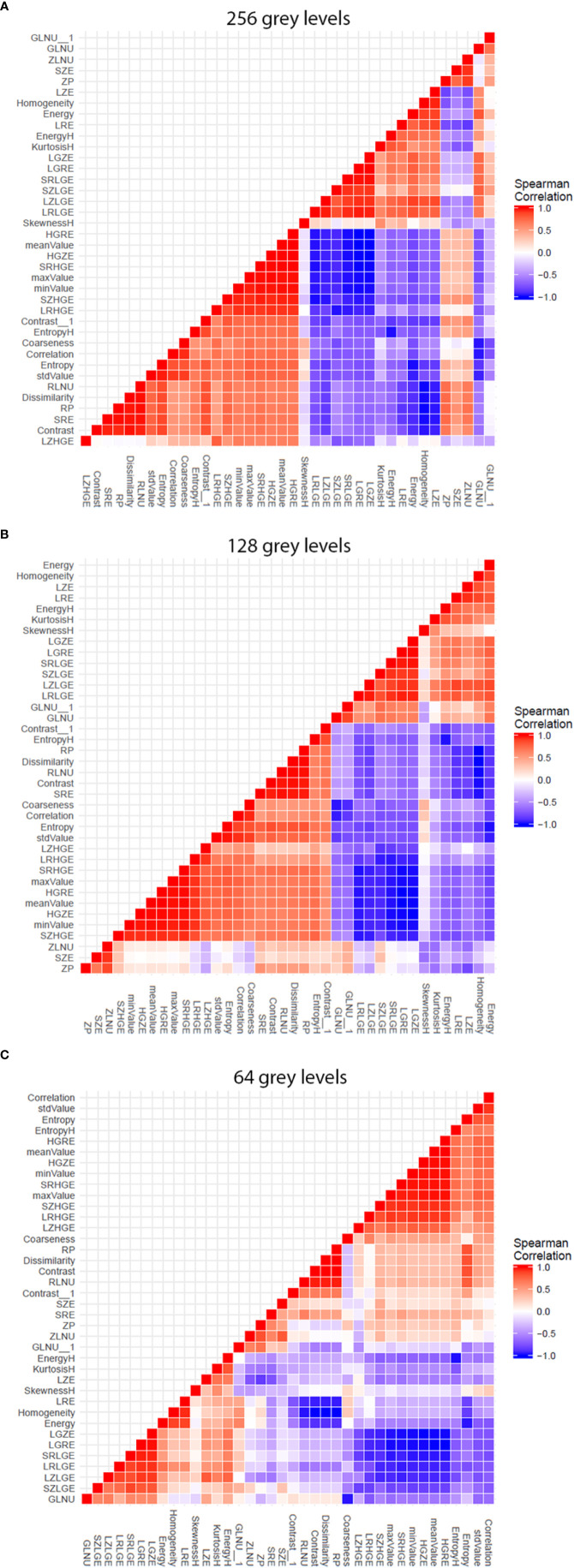
Graphs showing influence of software resampling step on phantom study. Each graph representes the two-by-two coefficient correlation calculation between the texture parameters at one resampling level. The difference in resulting patterns shows the influence of the software resampling step on texture parameters calculation, particularly on parameters extracted from co occurrence matrix. **(A)** 256 gray levels resampling. **(B)** 128 gray levels resampling. **(C)** 64 gray levels resampling.

The analysis software needs to resample the images gray scale by sub-sampling the number gray levels, in order to be able to calculate texture features. Thus, we firstly explored the influence of this sub-sampling step before exploring the influence of magnetic field strength. We did this through the two-by-two coefficient correlation calculation between the texture features (**Figure 2**). A colored (red/blue) hierarchical clustering is presented, showing the importance of the correlation beteween coefficients (**Figure 2**) for each level of resampling. A strong correlation was observed for a large number of texture features at the 256 gray level (only 9 features were totally independent with a Spearman’s rho <0,70). The correlation value decreased with the sub-sampling in gray levels resolution. The gray level sub-sampling influence on texture features was not significant for parameters extracted from the histogram. In contrast, the gray levels sub-sampling had a significant impact on parameters extracted from matrices.

#### Influence of Field Strength on Homogeneous Phantom

**Figure 3:**
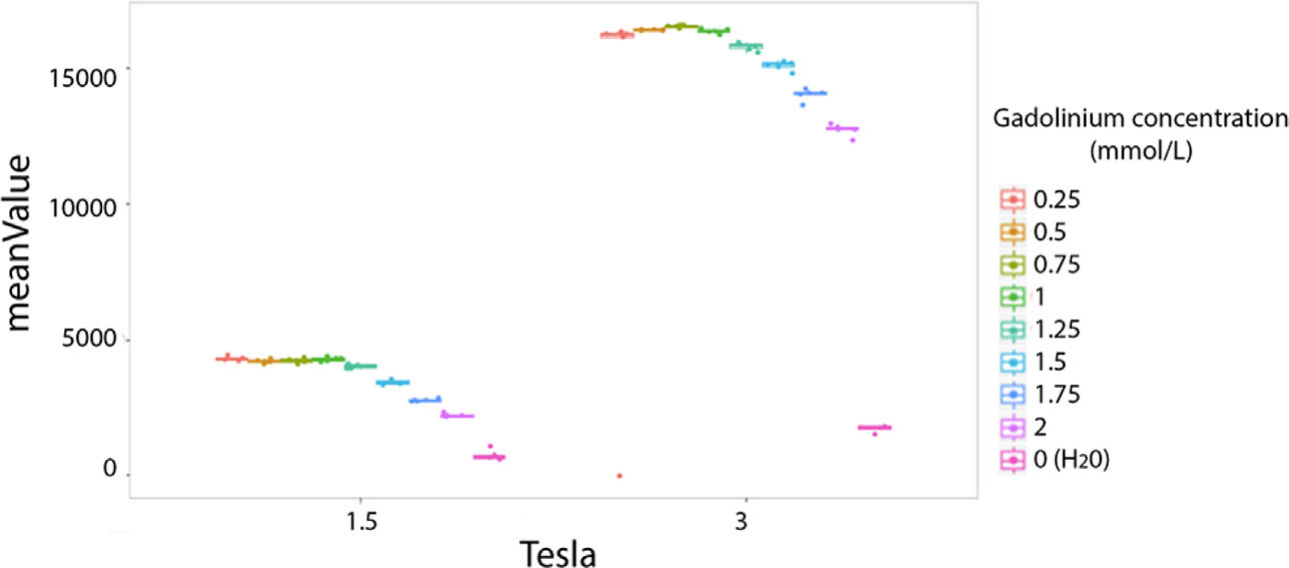
Difference of phantoms textures features values at 1.5 and 3T. Example of the mean value. Each colored dot groups shows the mean value calculated from repeated phantom MRI acquisition (one dot per MRI acquisition) in different homogeneous tubes with different concentration of Gadolinium chelate (on color per tube), respectively at 1.5 and 3T.

The majority of texture features values were significantly different between the two magnetic fields (1.5T vs. 3T). For example the mean value is presented for comparison between the two fields strenght (**Figure 3**). The entropy mean value was higher on 3T versus 1.5T by a factor of four (**Figure 3**).

#### Influence of Field Strength on Heterogeneous Phantoms

The majority of texture features were significantly different between the two magnetic fields (1.5T vs. 3T) according to the Student’s paired t-test (**Table 2**). Concerning features from histogram analysis, only Kurtosis, Entropy, and Energy did not significantly differ beteween 1.5 and 3T. For the matrix-based texture features, only LZHGE shows no significant difference.

**Table 2:** Texture features presenting significant or non-significant differences at 1.5 and 3T based on the heterogeneous phantoms examination (student’s paired t-test).

Significant difference in texture features value at 1.5 and 3T (p-values < 10^-5^)	LGRE, Homogeneity, SZE, ZP, Contrast, LGZE, Correlation, RLNU Entropy, SRLGE, LRHGE, HGRE, mean value, SRHGE, LRE, GLNU, HGZE, GLNU, SRE, SZHGE, SZLGE, LRLGE, max value, Dissimilarity Contrast, RP, std value, LZE, ZLNU, skewness, H min, value Coarseness, LZLGE
Non-significant difference in texture features value at 1.5 and 3Tp-values > 0.005	Energy H, Entropy H, Kurtosis H, LZHGE, Energy H

#### Influence of Pixel Size

**Figure 4:**
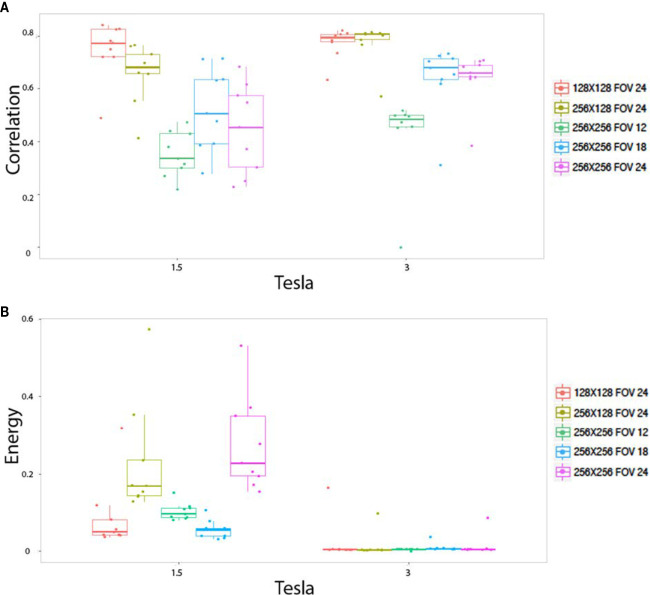
*In vitro* parameters value according to magnetic field strengh and pixel size: For correlation **(A)** and energy **(B)**. Texture features extracted to the homogeneous phantoms. Each colored dot groups and related box plot shows the value of correlation **(A)** and energy **(B)** calculated from repeated phantom MRI acquisition (one dot per MRI acquisition) with different pixel size (one color per matrix/FOV couple), respectively, at 1.5 and 3T.

The pixels size, dependant on both matrix size and FOV size, altered the radiomics output in homogeneous phantom according to three behaviors. First of all, pixel size altered the values of the texture features on both the 1.5 and 3T magnetic field, for 15 indices: stdvalue, skewness, Homogeneity, Contrast, Correlation, Entropy, Dissimilarity, ZLNU, GLNU, RLNU, Coarseness, SZE, LZE, GLNU_1, and ZP. **Figure 4A** illustrates the “Correlation” named feature. Second, pixel size altered the value of texture features only on 1.5T, but not on 3T: Kurtosis, Entropy H, Energy H, Energy, LRE, RP. **Figure 4B** illustrates “Energy” named feature. Note that in contrast “Contrast_1” named feature value was isolatedly modified only on 3T. Thirdly, pixel size did not significantly alter the value of texture features for: minvalue, meanvalue, maxvalu, HGRE, SRHGE, LRHGE, HGZE, SZHGE, and LZHE.

**Figure 5:**
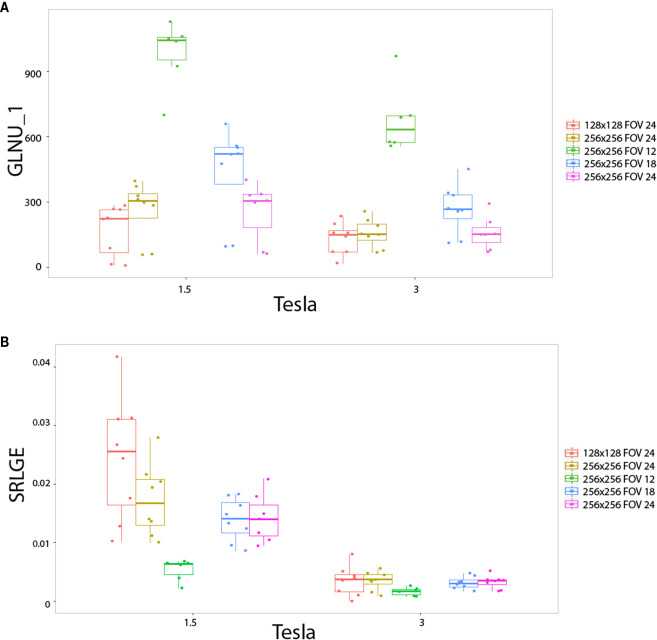
*In vitro* parameters value according to magnetic field strengh and pixel size: for GLNU_1 **(A)** and SRLGE **(B)**. Texture features extracted to the heterogeneous phantoms. Each colored dot groups and related box plot shows the value of GLNU_1 **(A)** and SRLGE **(B)** calculated from repeated phantom MRI acquisition (one dot per MRI acquisition) with different pixel size (one color per matrix/FOV couple), respectively, at 1.5 and 3T.

The impact of matrix size and FOV as well as pixel size, on the radiomics output was also studied with the heterogeneous phantoms. Also three behaviors were observed. First, pixel size altered the values of the texture features on the two magnetic fields for: RLNU, Coarseness, SZLGE, GLNU and ZLNU. **Figure 5A** shows the example of “GLNU” named feature. Second, pixel size altered the value of texture features only on 1.5T, but not on 3T for: Correlation, LGRE, SRLGE, GLNU, and LGZE. **Figure 5B** shows an example with the “SRLGE” named feature. Third, pixel size did not significantly alter the value of texture features for the other 29 texture parameters.

Ability of texture features to identify a difference between phantom tubes, in both field sthrengh separatly, regardless of the pixel size, is observed through the absolute Spearman’s rho correlation coefficient (**Table 3**). Eight imaging features identified the (visually obvious) difference between homogeneous and heterogeneous phantoms, with an absolute Spearman’s rho correlation coefficient above 0.5. The following texture features identified the difference between the different heterogeous phantoms (mainly different according to their polystyrene beads size and spatial distribution) in both field sthrengh separatly: dissimilarity, LZHGE, entropy, homogeneity, SRE, SZE, LRE, GLNU, RLNU, ZLNU, KurtosisH, LZE, entropyH, coarseness, SRLGE, SRLGE, SZLGE, and LGRE. Two texture features (coarseness, RLNU) identified the difference between homogeneous tubes (mainly different by their Gadolinium chelate concentration). As long as the pixel size remains sufficiently tiny small (1 mm or less in our study), 26 features were sensitive to texture alteration.

**Table 3:** Tabulation of textures features and their ability to dissociate the different phantom’s tubes at both field strengths separately, regardless to pixel size.

Heterogeneous vs. Homogeneous media		Size of polystyrene		Gadolinium concentration	
RLNU	0.796	Contrast	0.801	Coarseness	0.577
Coarseness	0.795	Contrast_1	0.795	RLNU	0.513
minValue	0.683	Dissimilarity	0.794	minValue	0.482
GLNU 1	0.617	LZHGE	0.789	GLNU	0.414
SZLGE	0.552	stdValue	0.786	SZLGE	0.371
SRLGE	0.534	Entropy	0.783	SRLGE	0.354
Entropy	0.645	Homogeneity	0.773	GLNU	0.347
Homogeneity	0.622	ZP	0.77	EntropyH	0.33
ZLNU	0.491	SRE	0.768	meanValue	0.328
EntropyH	0.484	RP	0.762	LGZE	0.325
LGRE	0.481	Energy	0.756	LGRE	0.315
LGZE	0.477	SZE	0.756	EnergyH	0.308
GLNU	0.468	LRE	0.741	KurtosisH	0.302
Contrast	0.458	GLNU	0.738	SkewnessH	0.293
EnergyH	0.439	RLNU	0.732	Correlation	0.262
Contrast	0.429	minValue	0.727	maxValue	0.246
Dissimilarity	0.423	ZLNU	0.698	Contrast	0.246
stdValue	0.421	KurtosisH	0.677	ZLNU	0.237
Energy	0.418	LZE	0.646	LRLGE	0.228
meanValue	0.409	EntropyH	0.624	LRHGE	0.198
KurtosisH	0.405	Coarseness	0.617	Entropy	0.195
RP	0.378	SRLGE	0.583	Energy	0.182
SRE	0.373	EnergyH	0.554	Contrast	0.181
Correlation	0.363	SZLGE	0.554	Dissimilarity	0.18
SZE	0.346	LGRE	0.524	LZLGE	0.173
LRE	0.329	LGZE	0.46	Homogeneity	0.165
ZP	0.329	LRHGE	0.373	stdValue	0.155
LRHGE	0.316	LZLGE	0.321	HGZE	0.15
LRLGE	0.3	meanValue	0.275	HGRE	0.145
SkewnessH	0.229	SkewnessH	0.267	RP	0.141
maxValue	0.228	LRLGE	0.244	SZHGE	0.137
LZHGE	0.21	maxValue	0.112	SRE	0.132
HGZE	0.185	HGRE	0.101	SRHGE	0.132
HGRE	0.174	GLNU	0.092	SZE	0.132
SRHGE	0.14	HGZE	0.087	LRE	0.111
SZHGE	0.132	SRHGE	0.041	ZP	0.104
LZLGE	0.097	SZHGE	0.037	LZE	0.077
LZE	0.096	Correlation	0.017	LZHGE	0.01

Given are the names of the feature variables and the absolute Spearman’s rho correlation coefficient between imaging features and the type of phantom tube. First column: ability to dissociate homogeneous versus heterogeneous tubes. Second column: ability to dissociate heterogeneous tubes according to their polystyrene beads sizes and their spatial distribution. Third column: ability to dissociate homogeneous tubes according to their Gadolinium chelate concentration.

### Effect of Field Strength on MRI Texture Features Values in Healthy Volunteers

**Figure 6:**
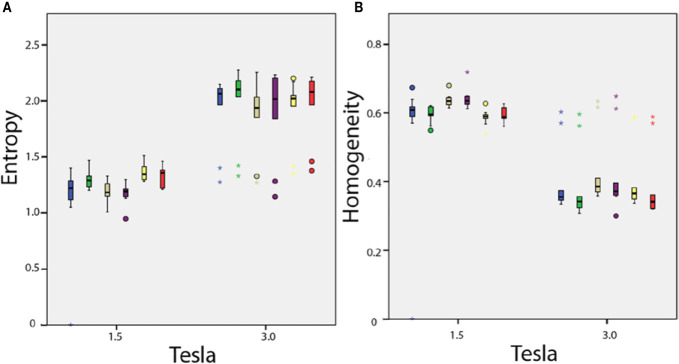
*In vivo* parameters value difference according to magnetic field strength: **(A)** entropy and **(B)** homogeneity. Each box plot represents the values dispersion of the 10 healthy volunteers for one of the six anatomical regions.

Among the 38 parameters, significant differences were observed with 15 texture features measured in healthy volunteers between 1.5 and 3T MRIs for the same anatomical region (**Figure 6**). With constant fields (1.5T vs. 1.5T and 3T vs. 3T), those 15 texture features values appeared identical when measured in symmetrical anatomical structures (eg corpus calosum or caudate nucleus).

## Discussion

Our study demonstrates that field strength (1.5T vs. 3T) influences numerous texture features values for both phantom and human exploration. Our study also demonstrated that gray-level resampling and pixel size influence some texture features. Those results are of importance for clinical study design and for patient examination and follow-up. In addition to that, our study identifies texture parameters that are able to differentiate phantom textures in our setup. It is important to mention that these relevant textures parameters are significantly influenced by field strength.

An increasing number of radiomics or deep-learning studies use imaging based on MRI acquisitions (24–28). Confounding parameters that can alter radiomic output have been largely addressed for CT scans but less for MRIs (27, 29–31). Our study aims to complete knowledge in the field of radiomics applied to neuroradiology MRI acquisitions.

The presented study identifies the importance to consider the impact of magnetic field strength on radiomics features values in clinical practice. SNR is well-known to increase when the field strength increases. We hypothesize that our result may be partially due to this difference in SNR between the two MRI machines. Even given that field strength is a major factor influencing signal intensity, further factors modifying signal have to be considered, as discussed in a recent literature review. Mayerhoefer et al. previously demonstrated that MRI texture features are influenced by change in SNR (5, 9). The difference in SNR could be explained by magnetic field strength, but SNR is also linked to the entire signal acquisition system (coils, electronic device etc.). The European research project COST B11 (31, 32), aimed at developing quantitative methods for MRI imaging features extraction (between 1998 and 2002), demonstrated that signal and subsequently texture features were dependent on parameters such as configuration of the transmitting and/or receiving RF coil (antenna), and the number of active segments in the coil due to change in tilt angle.

Our results showed that most texture features are also sensitive to the variations in matrix and FOV and consequently spatial resolution, even if the clinical ranges in matrix size and FOV for a dedicated organ may be thinner than those investigated in literature physics studies. Of note, we acquired images at different pixel sizes whereas most studies changed the pixel size during post-processing. Our study showed that as long as the pixel size remains sufficiently small (1 mm in our experience), 26 features are sensitive to texture alteration. This is in accordance with Jirák study. which also compared MRI features measured on phantoms with nodular patterns in a multicenter settings and showed that texture classification was influenced by low resolution causing large errors (10).

For a defined magnetic field strength, our study showed that part of the texture features differentiated the heterogeneous phantoms from the homogeneous phantoms on all sequences, irrespective of the pixel size, and part of radiomics features differentiated phantoms with large and small spherical objects (polystyrene beads) scattered in agarose gel. These results are in agreement with literature (8, 10, 28, 29, 33, 34).

In our study, gray level resampling has a significant impact on radiomic parameters. This observation is in accordance with literature. Collewet et al. resampled gray scale using four methods: original gray levels, same maximum for all images, same average for all images, and limited dynamics. Results also showed the influence of the normalization method on the texture classification accuracy.

While our study focuses on field strength, pixel size and gray level resampling, previous studies have identified further MRI sequence parameters of interest, including repeat time (TR), echo time (TE) and receiver bandwidth (BW). Mayerhoefer et al. evaluated how texture features varied according to acquisition parameters (e.g., TR, TE, sampling bandwidth [SBW], and spatial resolution). Interestingly, variations in TR, TE and SBW had little effect on pattern discrimination results, as long as a high-spatial resolution was observed. In addition, both studies (Mayerhoefer’s and ours) showed higher stability with the texture features derived from the co-occurrence matrix over first order texture features for characterizing patterns close to the resolution limits in varying conditions of TR/TE or pixel size.

Our approach to evaluate measures of radiomic features on standardized phantoms modeled to the organ under investigation is in line with the study by Bianchini et al. who explicitely designed and used an organ specific phantom for the optimization of radiomic studies of the female pelvis (35).

The question of the stability of our phantoms during the delay between the different acquisition/machine can be raised. Jirák et al. evaluated the long-term stability of the agarose phantoms, the optimal choice of texture parameters, and compared different MRI magnetic fields (3T, 4T, and 7T) (8, 10). They demonstrated that phantoms are stable over 12 months. In our study, all sequences were acquired on the same day within the same hour of examination, consequently we did not expect to incur phantom stability issue in our setup.

Optimal methodological guidelines are currently being defined to optimize data analysis strategies (4, 36–38) in the field of radiomics. Our results are critical since they demonstrate that standardization strategies for the use of radiomics in MRI should focus on addressing the challenge of heterogeneity in protocols since there is a dramatical difference between radiomics features extracted from the same patient or the same phantom at 1.5 or 3T.

Our study demonstrates that field strength had a strong influence on texture feature values, also spatial resolution and gray scale resampling. Quantification of the impact of the various parameters of imaging features is of major interest. Our findings are clinically relevant as image acquisition was performed according to daily used protocols for clinical examinations. Those confounding factors need to be adjusted when designing a multicentric trial and when adapting the results of a study to different platforms.

## Data Availability Statement

The datasets generated for this study are available on request to the corresponding author.

## Ethics Statement

This study was approved by the Scientific Review Committee of Gustave Roussy.

## Author Contributions

All authors contributed to the article and approved the submitted version.

## Conflict of Interest

The authors declare that the research was conducted in the absence of any commercial or financial relationships that could be construed as a potential conflict of interest.

## Abbreviations

3D SPGR, 3D rapid gradient echo sequence; EORTC, European Organization for Research and Treatment of Cancer; TR, Repeat time; MTX, Image matrix; CoMat, The co-occurrence matrix; GRLM, The gray-level run length matrix; GZLM, The gray-level zone length matrix; SBW, Sampling bandwidth.
