# Supplementary material for: Influence of Magnetic Field Strength on Magnetic Resonance Imaging Radiomics Features in Brain Imaging, an In Vitro and In Vivo Study
Source: Front Oncol. 2021 Jan 20;10:541663. doi: 10.3389/fonc.2020.541663 (PMC7855708; doi:10.3389/fonc.2020.541663)
Supplement: Supplementary file 1 [file DataSheet_1.pdf]

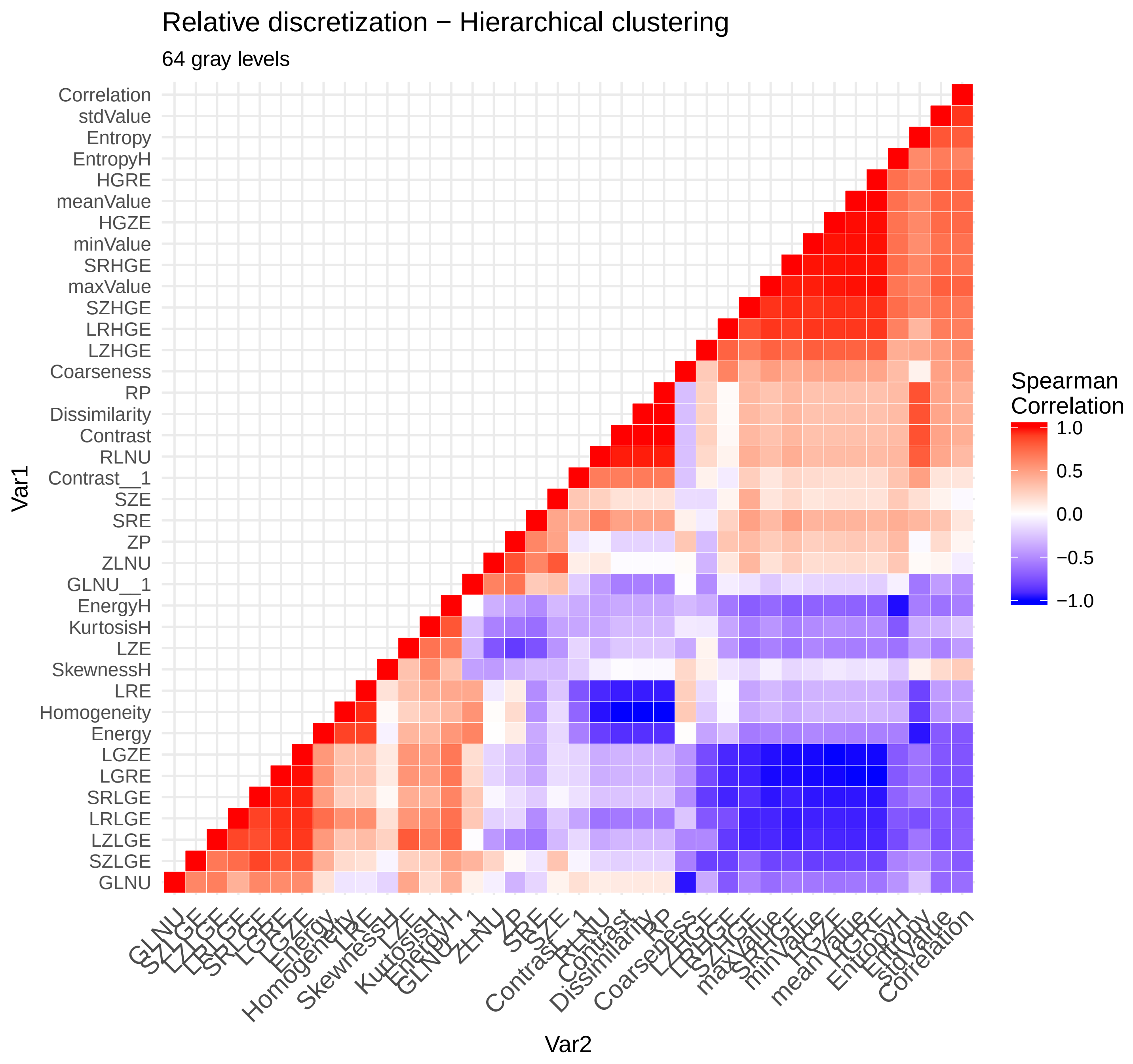
Relative discretization − Hierarchical clustering
64 gray levels
Var1
Var2
Correlation
stdValue
Entropy
EntropyH
HGRE
meanValue
HGZE
minValue
SRHGE
maxValue
SZHGE
LRHGE
LZHGE
Coarseness
RP
Dissimilarity
Contrast
RLNU
Contrast__1
SZE
SRE
ZP
ZLNU
GLNU__1
EnergyH
KurtosisH
LZE
SkewnessH
LRE
Homogeneity
Energy
LGZE
LGRE
SRLGE
LRLGE
LZLGE
SZLGE
GLNU
Spearman
Correlation
1.0
0.5
0.0
−0.5
−1.0

Relative discretization − Hierarchical clustering

128 gray levels

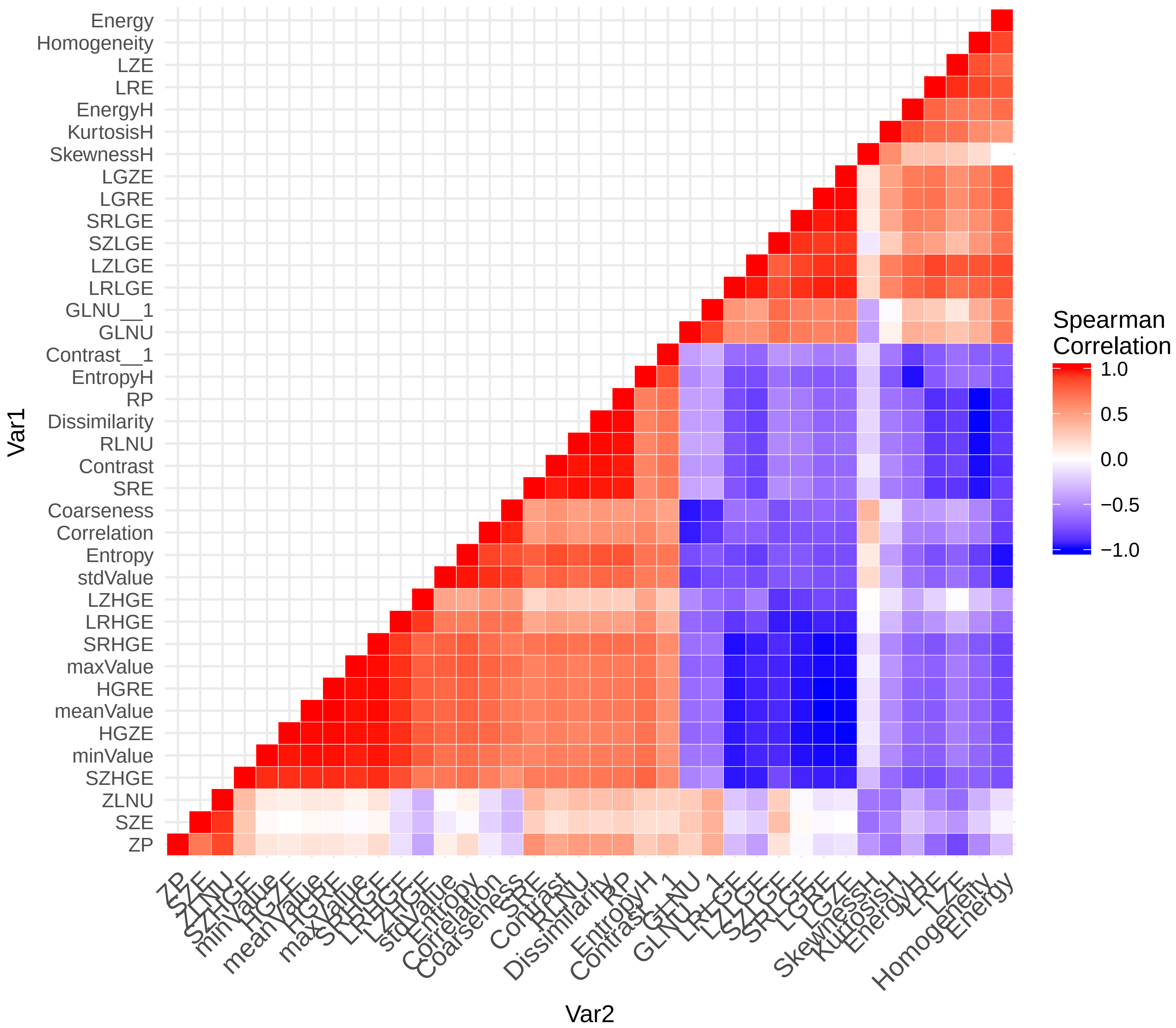

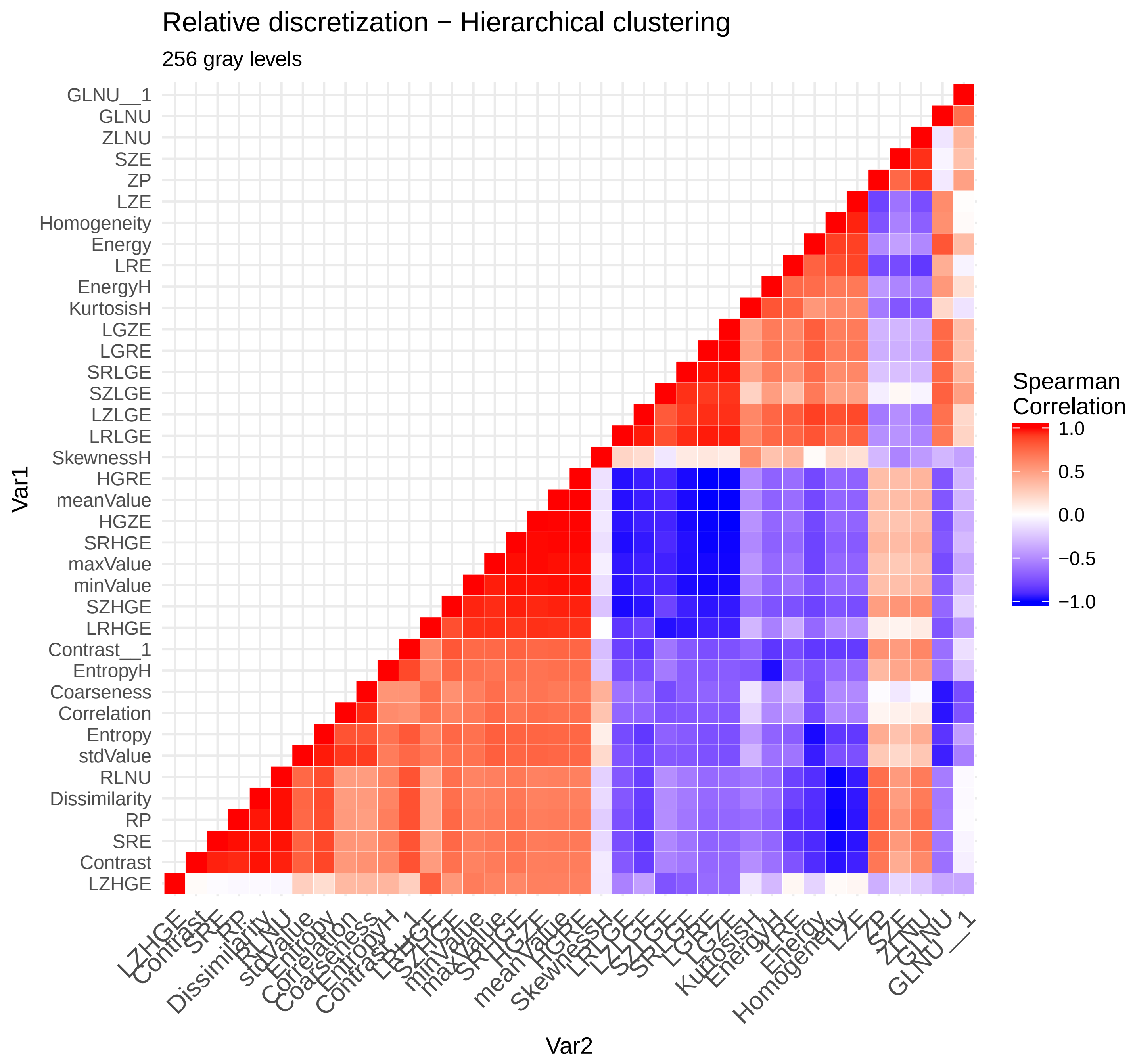
Relative discretization − Hierarchical clustering
256 gray levels
Spearman Correlation
1.0
0.5
0.0
−0.5
−1.0
Var1
Var2
GLNU__1
GLNU
ZLNU
SZE
ZP
LZE
Homogeneity
Energy
LRE
EnergyH
KurtosisH
LGZE
LGRE
SRLGE
SZLGE
LZLGE
LRLGE
SkewnessH
HGRE
meanValue
HGZE
SRHGE
maxValue
minValue
SZHGE
LRHGE
Contrast__1
EntropyH
Coarseness
Correlation
Entropy
stdValue
RLNU
Dissimilarity
RP
SRE
Contrast
LZHGE
