# Supplementary material for: Influence of Magnetic Field Strength on Magnetic Resonance Imaging Radiomics Features in Brain Imaging, an In Vitro and In Vivo Study
Source: Front Oncol. 2021 Jan 20;10:541663. doi: 10.3389/fonc.2020.541663 (PMC7855708; doi:10.3389/fonc.2020.541663)
Supplement: Supplementary file 2 [file DataSheet_2.pdf]

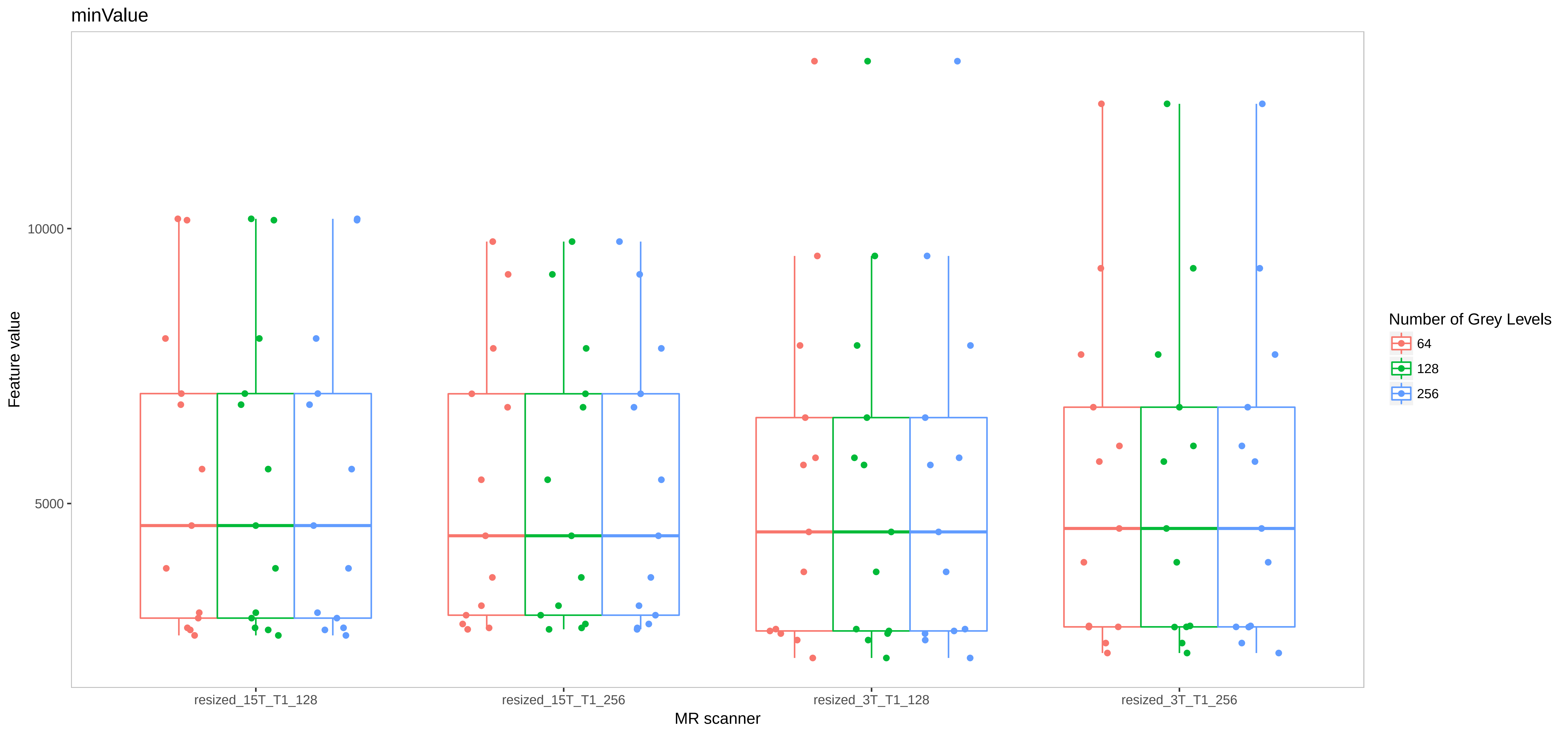
minValue
Feature value
10000
5000
resized_15T_T1_128
resized_15T_T1_256
resized_3T_T1_128
resized_3T_T1_256
MR scanner
Number of Grey Levels
64
128
256

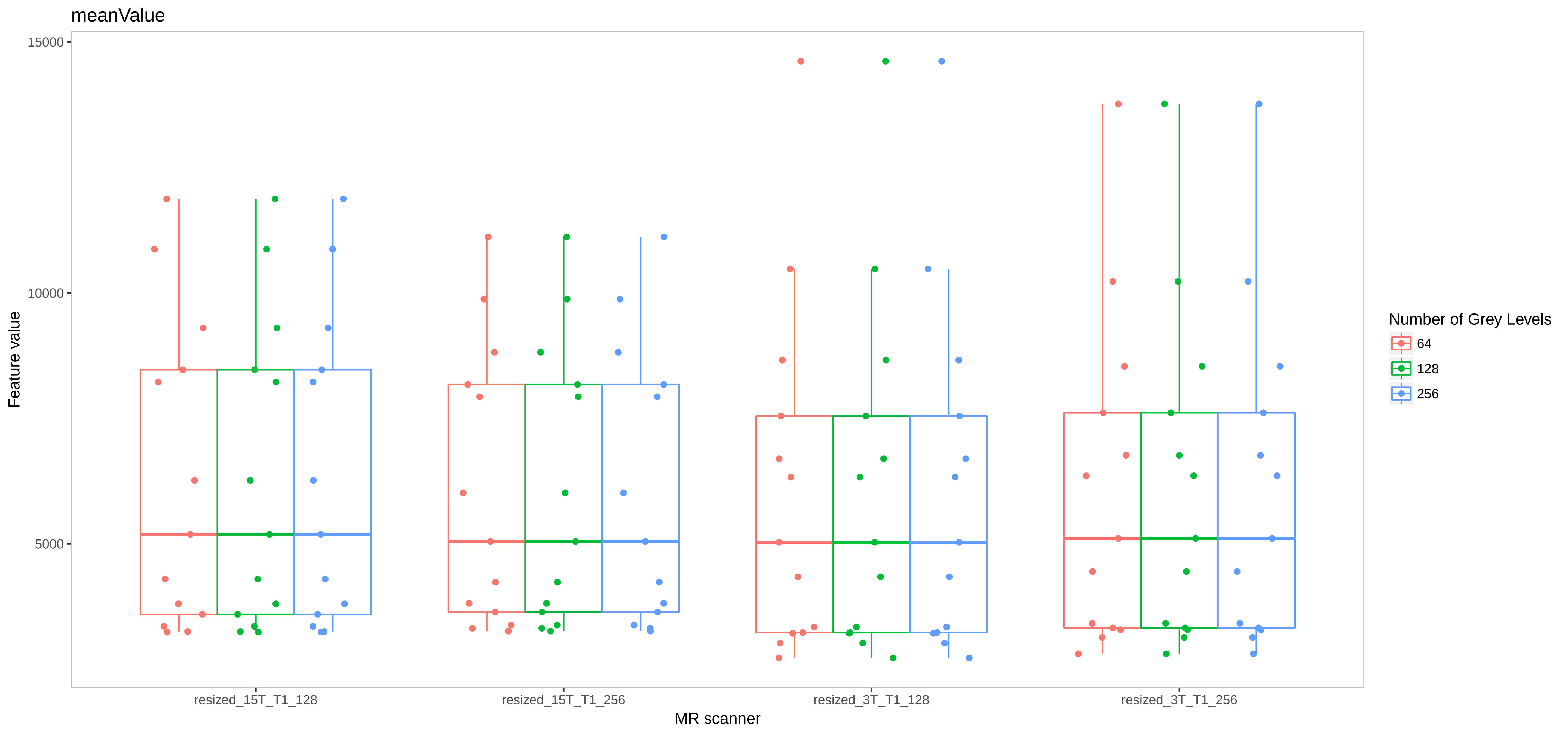

meanValue
15000
10000
5000
Feature value
resized_15T_T1_128
resized_15T_T1_256
resized_3T_T1_128
resized_3T_T1_256
MR scanner
Number of Grey Levels
64
128
256

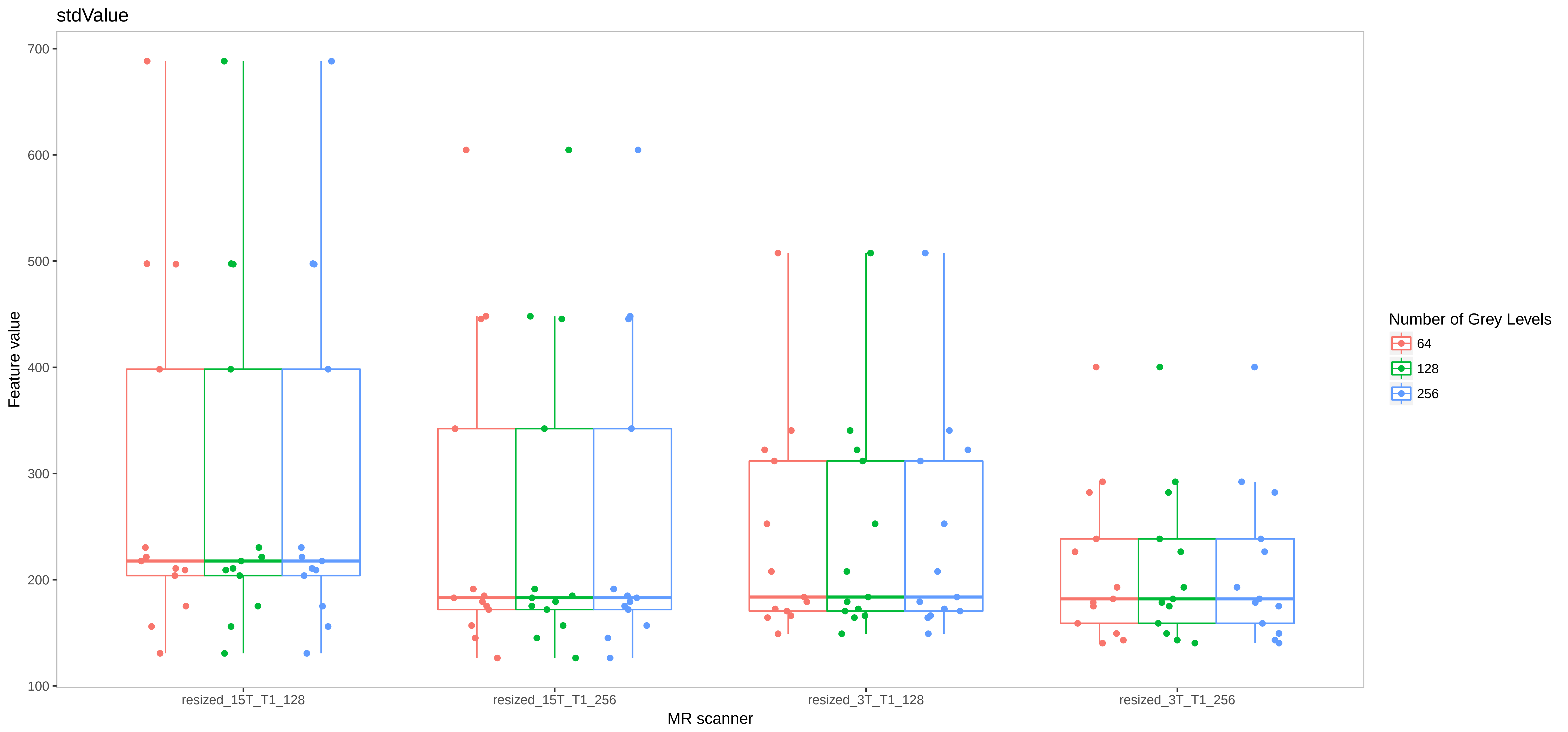
stdValue
Feature value
700
600
500
400
300
200
100
resized_15T_T1_128
resized_15T_T1_256
resized_3T_T1_128
resized_3T_T1_256
MR scanner
Number of Grey Levels
64
128
256

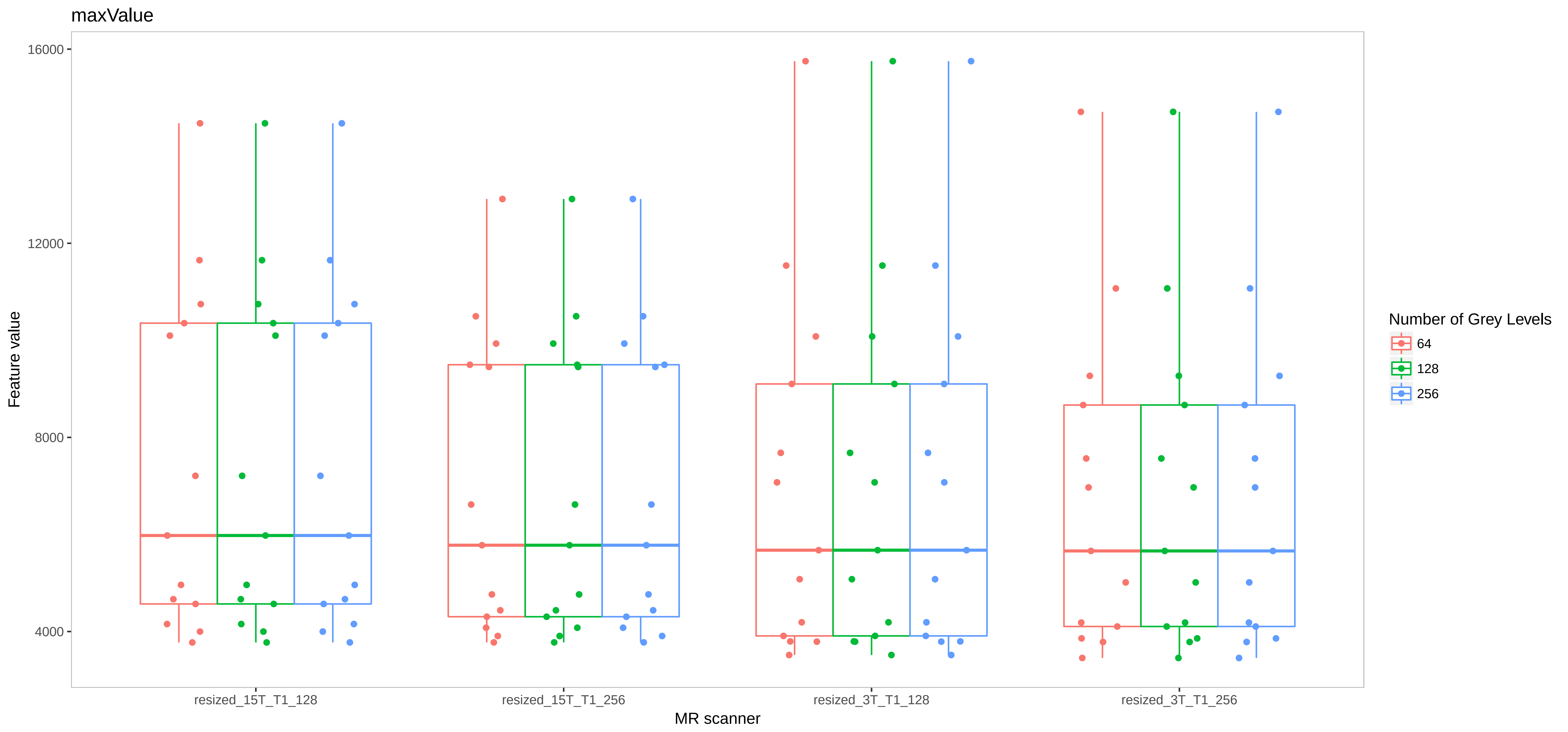

maxValue
16000
12000
8000
4000
Feature value
resized_15T_T1_128
resized_15T_T1_256
resized_3T_T1_128
resized_3T_T1_256
MR scanner
Number of Grey Levels
64
128
256

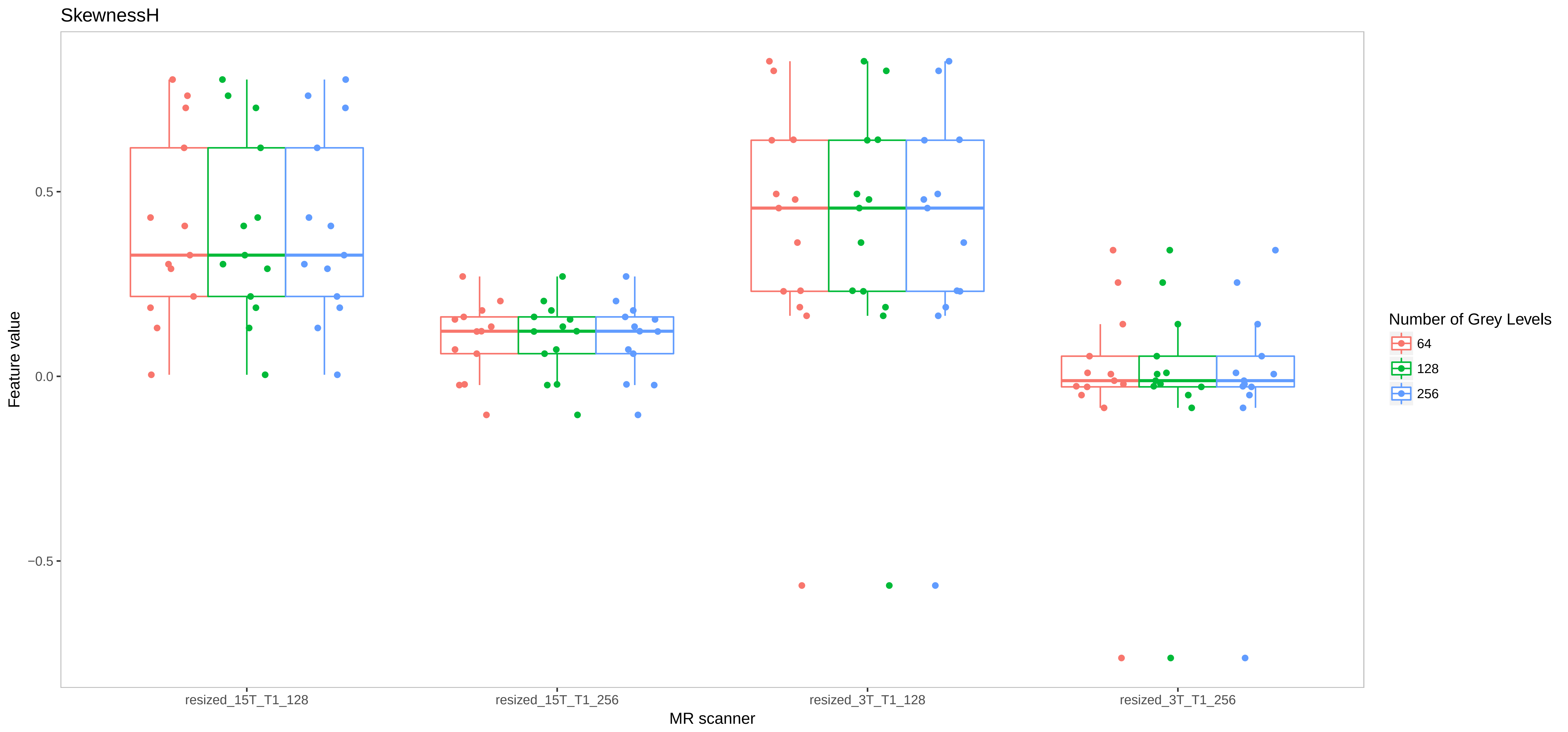

SkewnessH
Feature value
0.5
0.0
−0.5
resized_15T_T1_128
resized_15T_T1_256
resized_3T_T1_128
resized_3T_T1_256
MR scanner
Number of Grey Levels
64
128
256

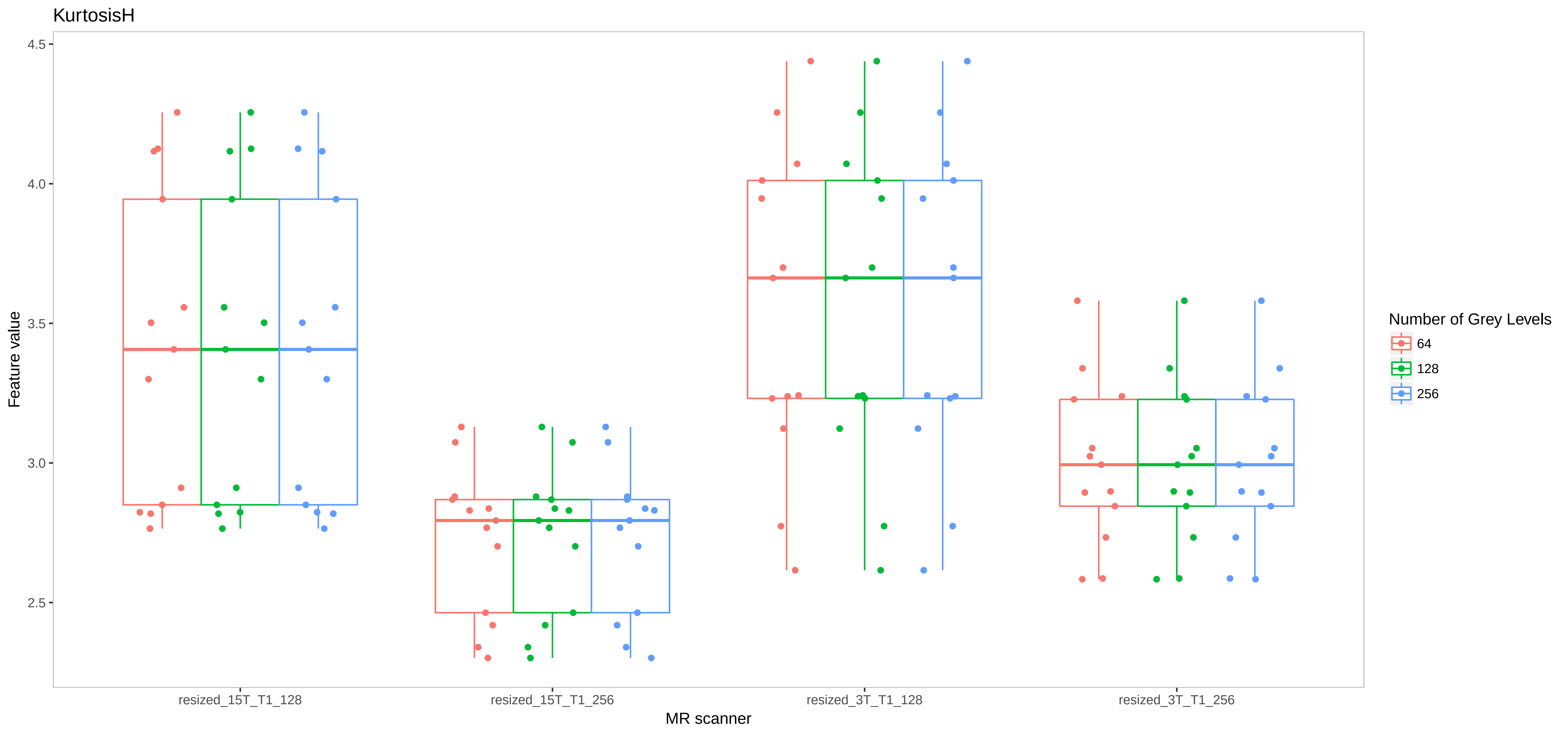
KurtosisH
Feature value
4.5
4.0
3.5
3.0
2.5
resized_15T_T1_128
resized_15T_T1_256
resized_3T_T1_128
resized_3T_T1_256
MR scanner
Number of Grey Levels
64
128
256

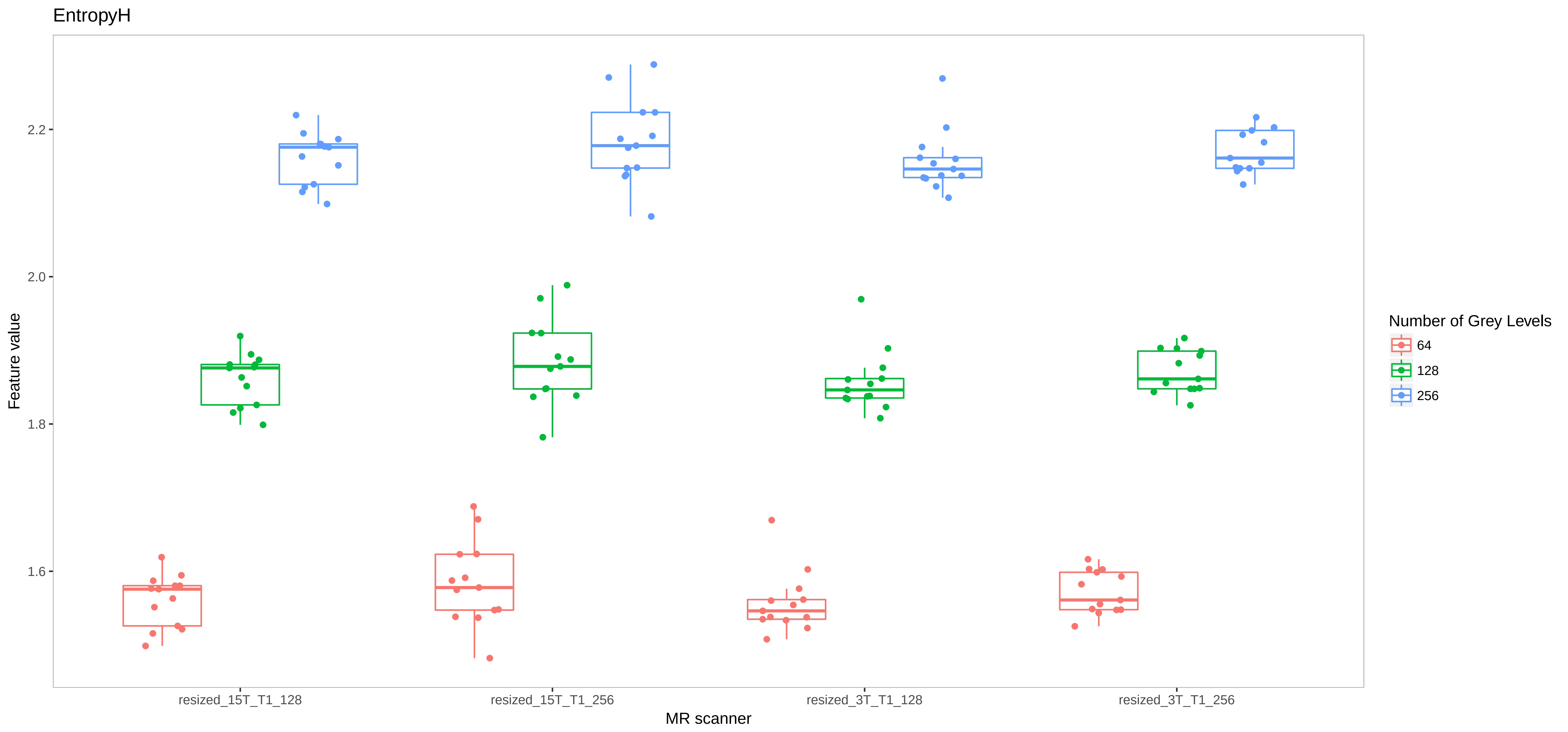
EntropyH
Feature value
2.2
2.0
1.8
1.6
resized_15T_T1_128
resized_15T_T1_256
resized_3T_T1_128
resized_3T_T1_256
MR scanner
Number of Grey Levels
64
128
256

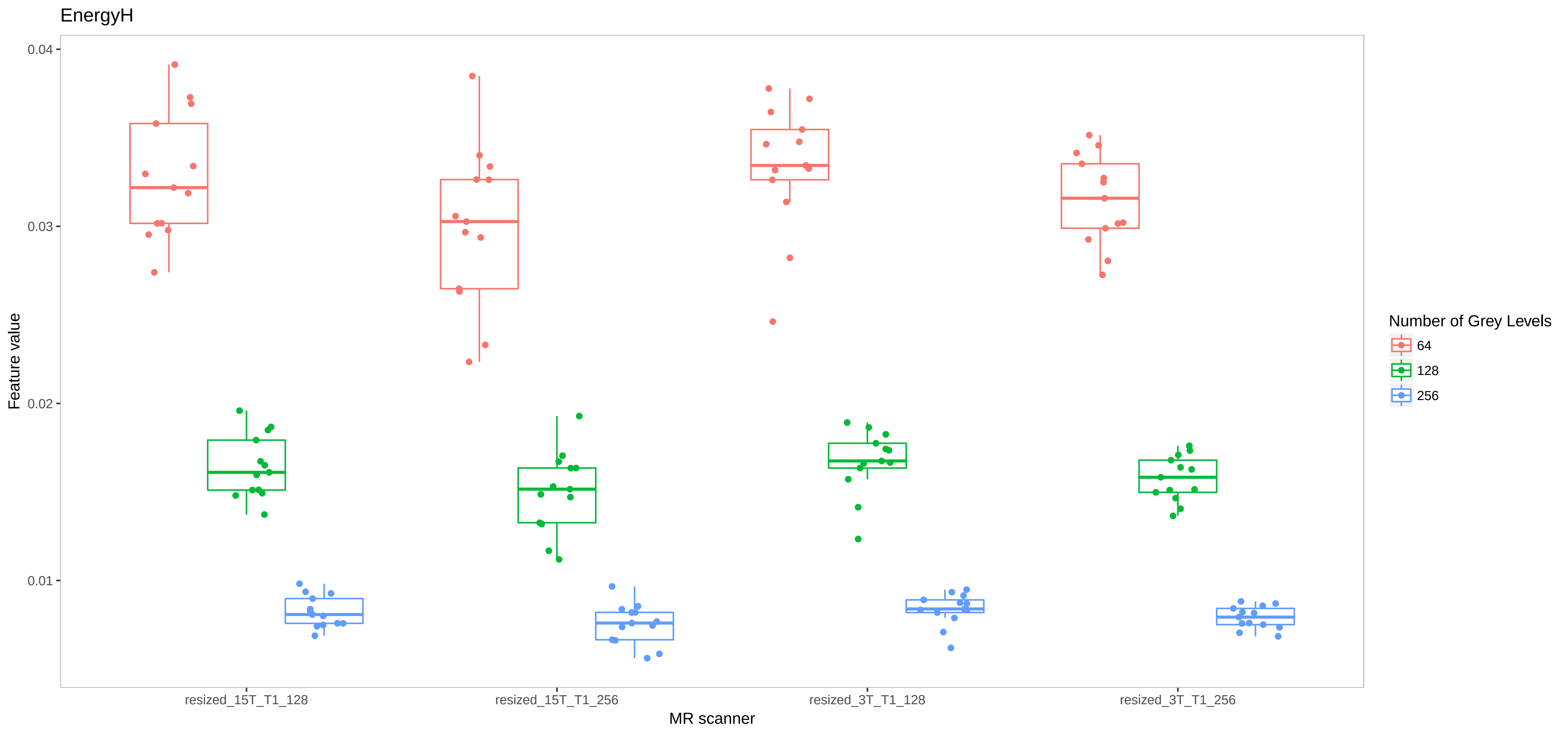
EnergyH
Feature value
0.04
0.03
0.02
0.01
resized_15T_T1_128
resized_15T_T1_256
resized_3T_T1_128
resized_3T_T1_256
MR scanner
Number of Grey Levels
64
128
256

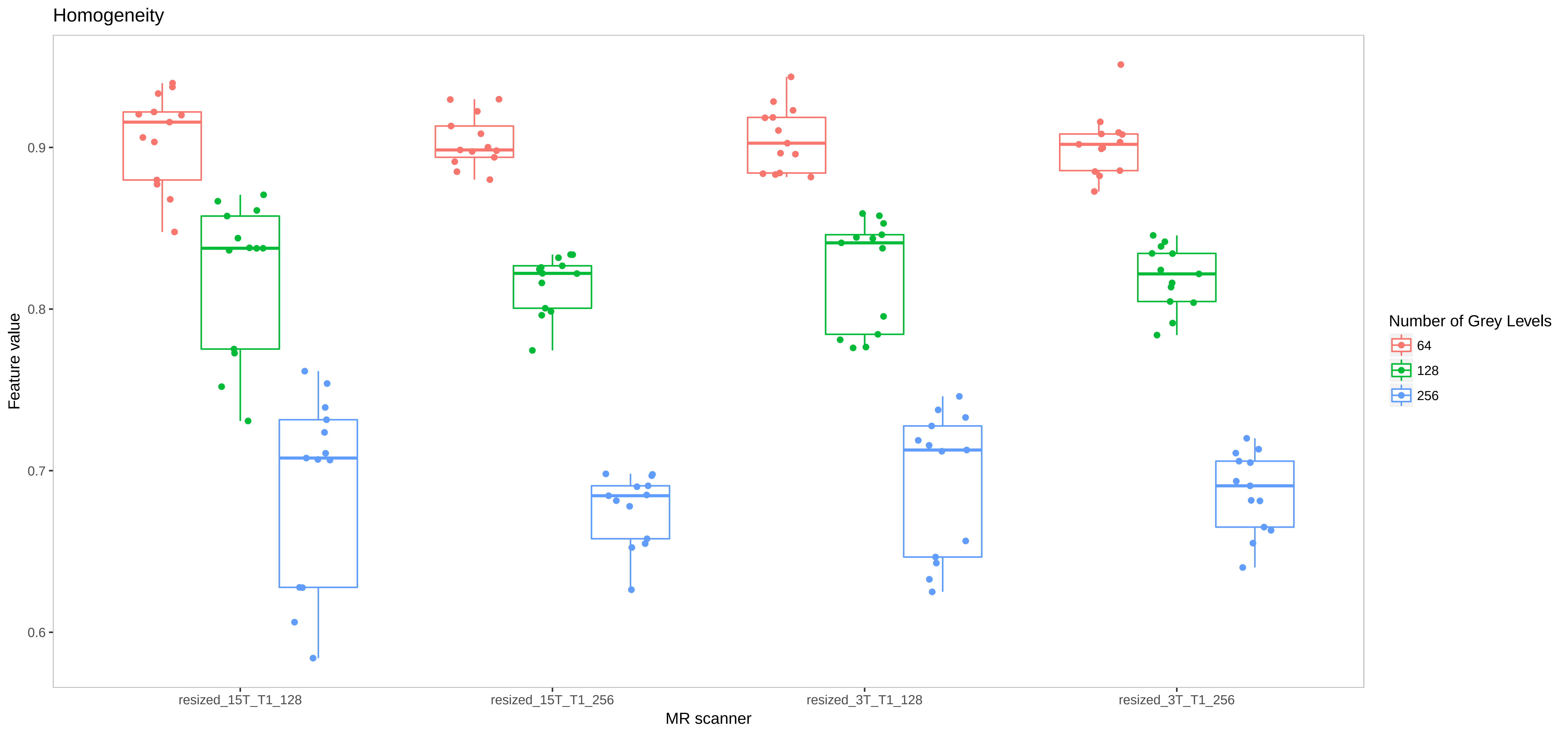

Homogeneity
Feature value
0.9
0.8
0.7
0.6
resized_15T_T1_128
resized_15T_T1_256
resized_3T_T1_128
resized_3T_T1_256
MR scanner
Number of Grey Levels
64
128
256

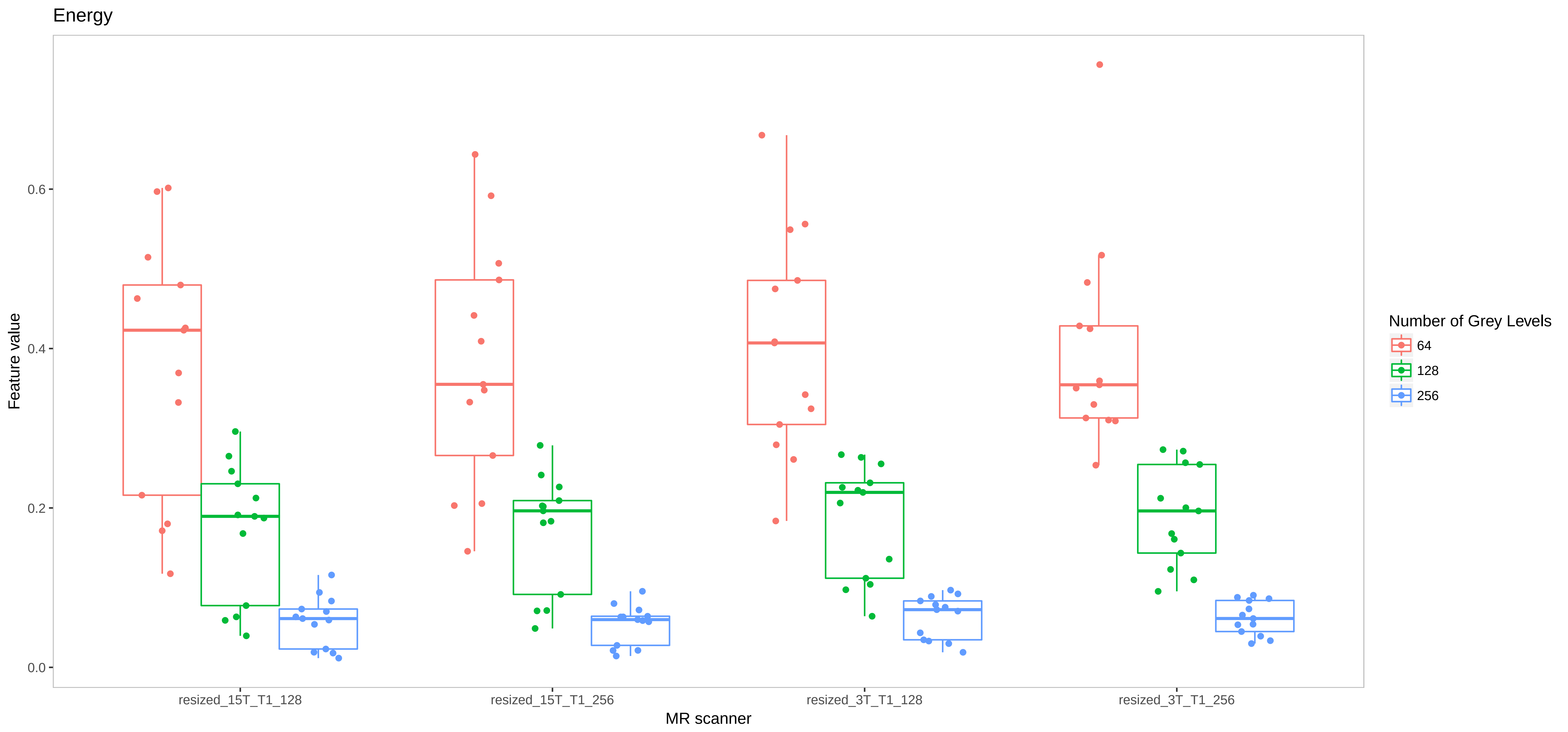
Energy
Feature value
0.0
0.2
0.4
0.6
resized_15T_T1_128
resized_15T_T1_256
resized_3T_T1_128
resized_3T_T1_256
MR scanner
Number of Grey Levels
64
128
256

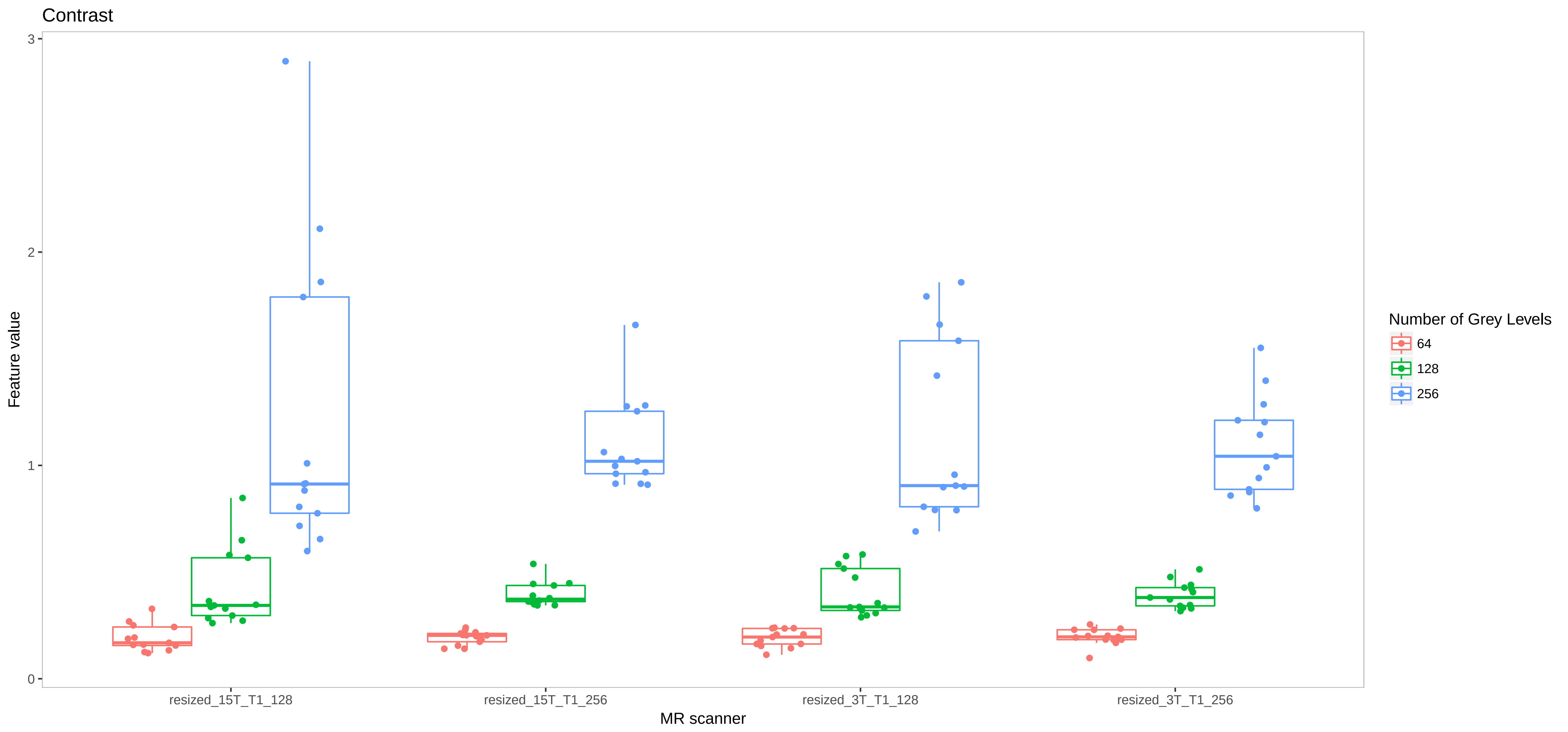

Contrast
Feature value
3
2
1
0
resized_15T_T1_128
resized_15T_T1_256
resized_3T_T1_128
resized_3T_T1_256
MR scanner
Number of Grey Levels
64
128
256

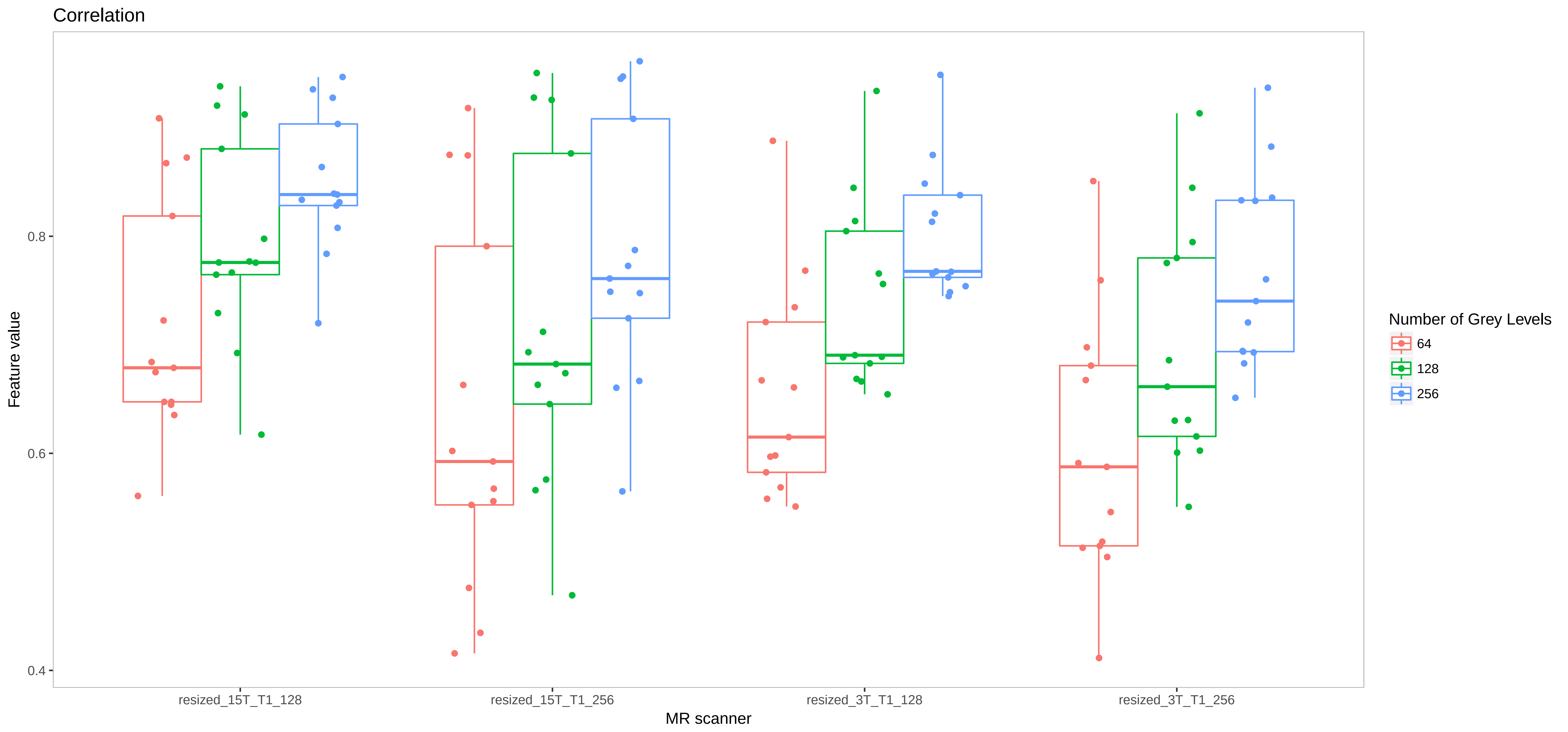
Correlation
Feature value
0.8
0.6
0.4
resized_15T_T1_128
resized_15T_T1_256
resized_3T_T1_128
resized_3T_T1_256
MR scanner
Number of Grey Levels
64
128
256

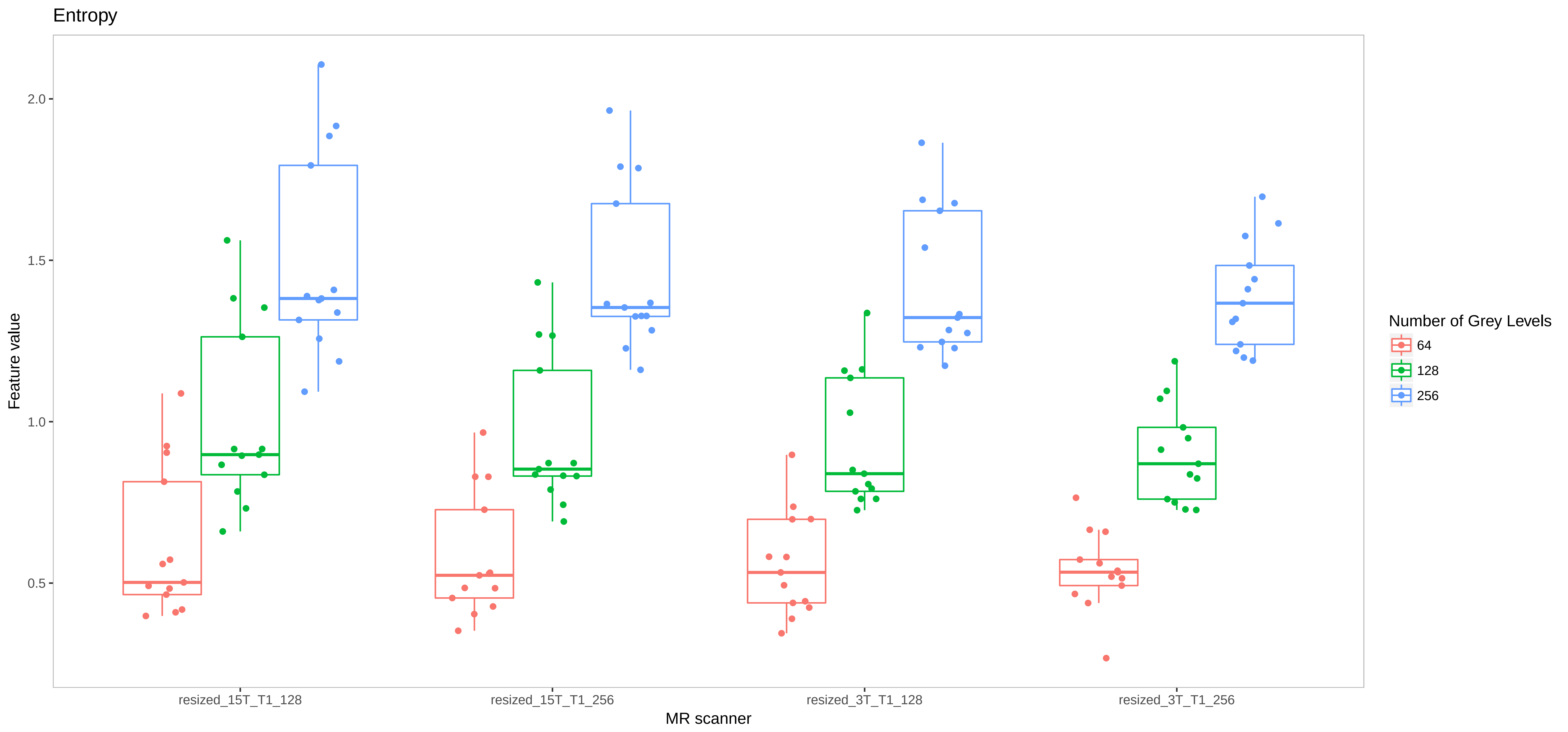

Entropy
Feature value
2.0
1.5
1.0
0.5
resized_15T_T1_128
resized_15T_T1_256
resized_3T_T1_128
resized_3T_T1_256
MR scanner
Number of Grey Levels
64
128
256

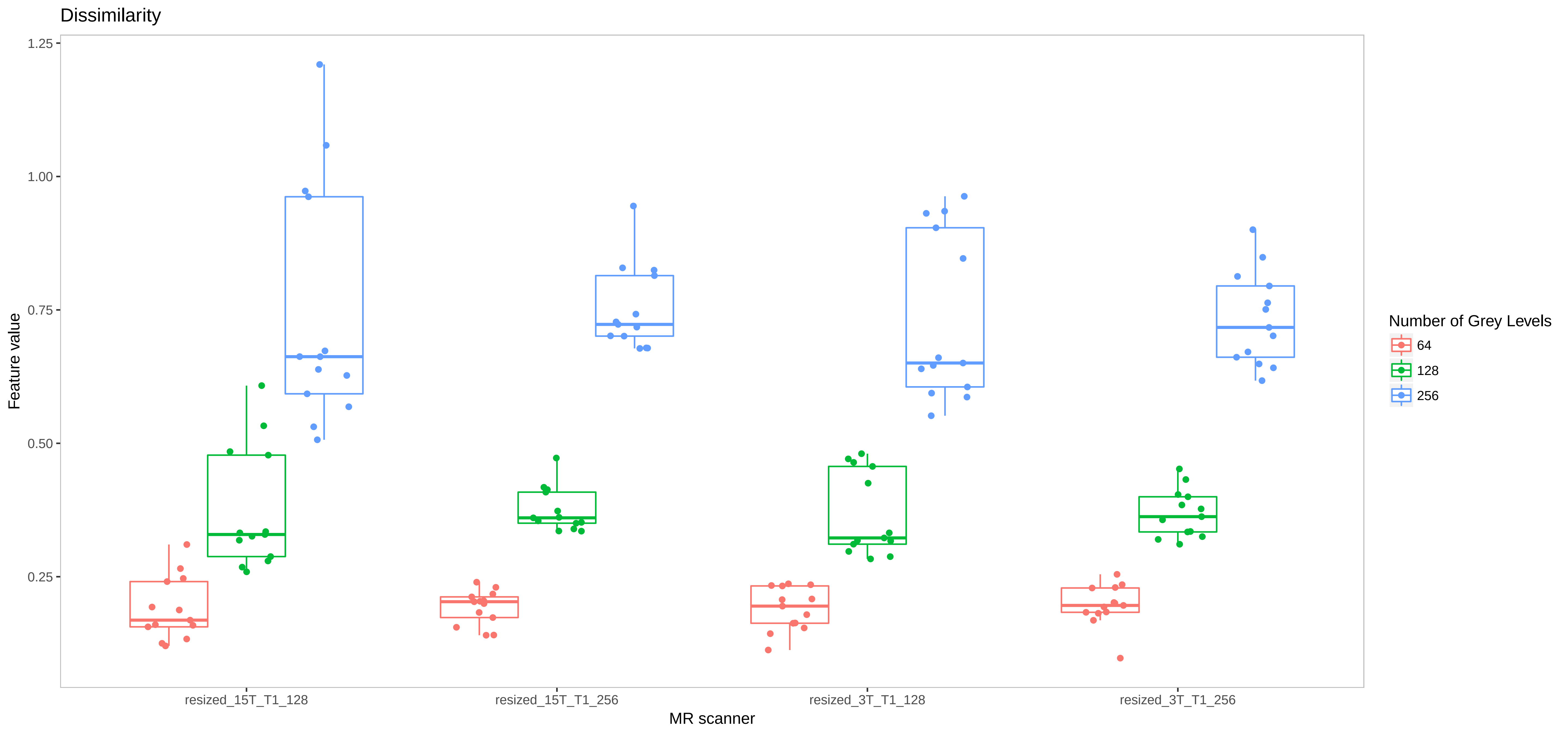
Dissimilarity
1.25
1.00
0.75
0.50
0.25
Feature value
resized_15T_T1_128
resized_15T_T1_256
resized_3T_T1_128
resized_3T_T1_256
MR scanner
Number of Grey Levels
64
128
256

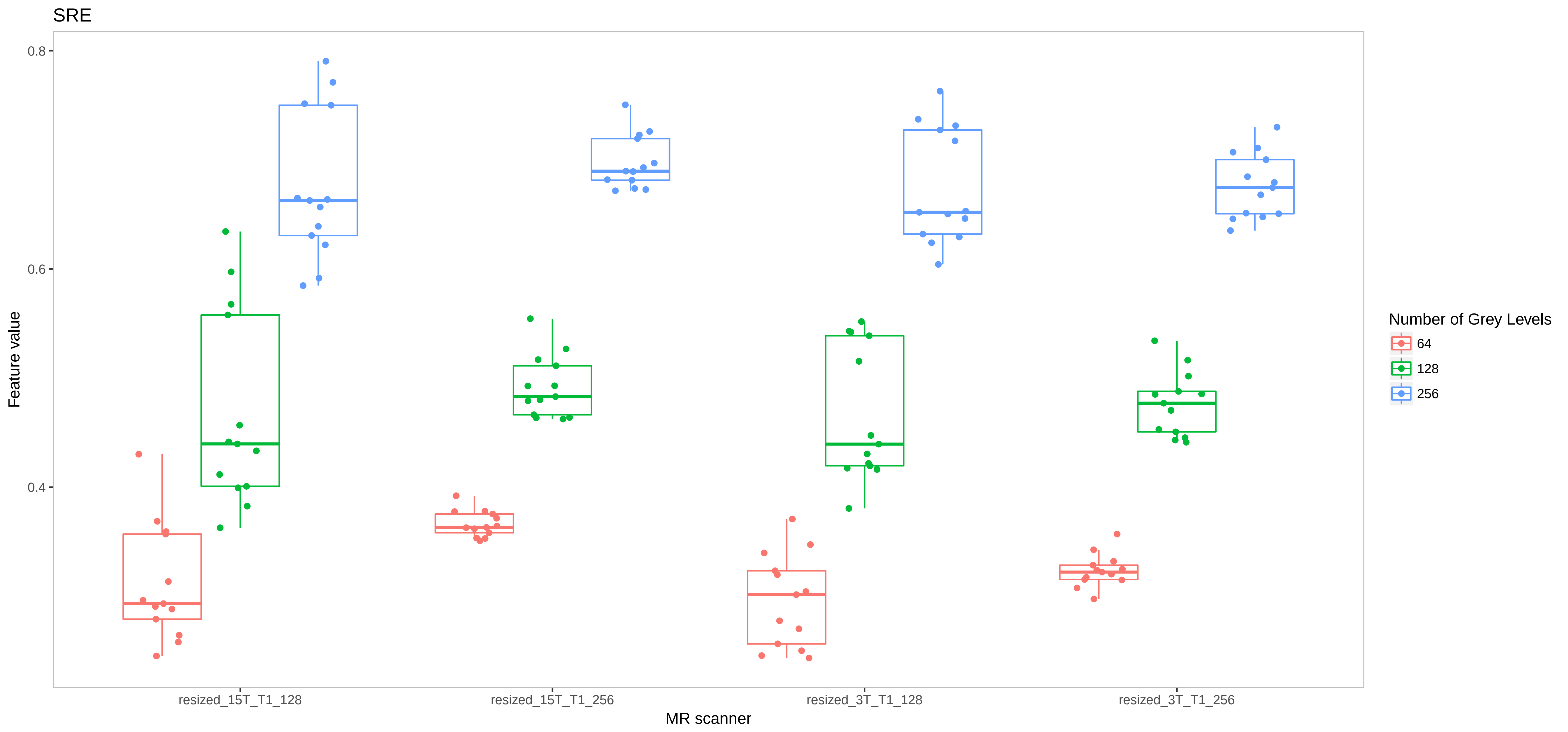

SRE
0.8
0.6
0.4
Feature value
resized_15T_T1_128
resized_15T_T1_256
resized_3T_T1_128
resized_3T_T1_256
MR scanner
Number of Grey Levels
64
128
256

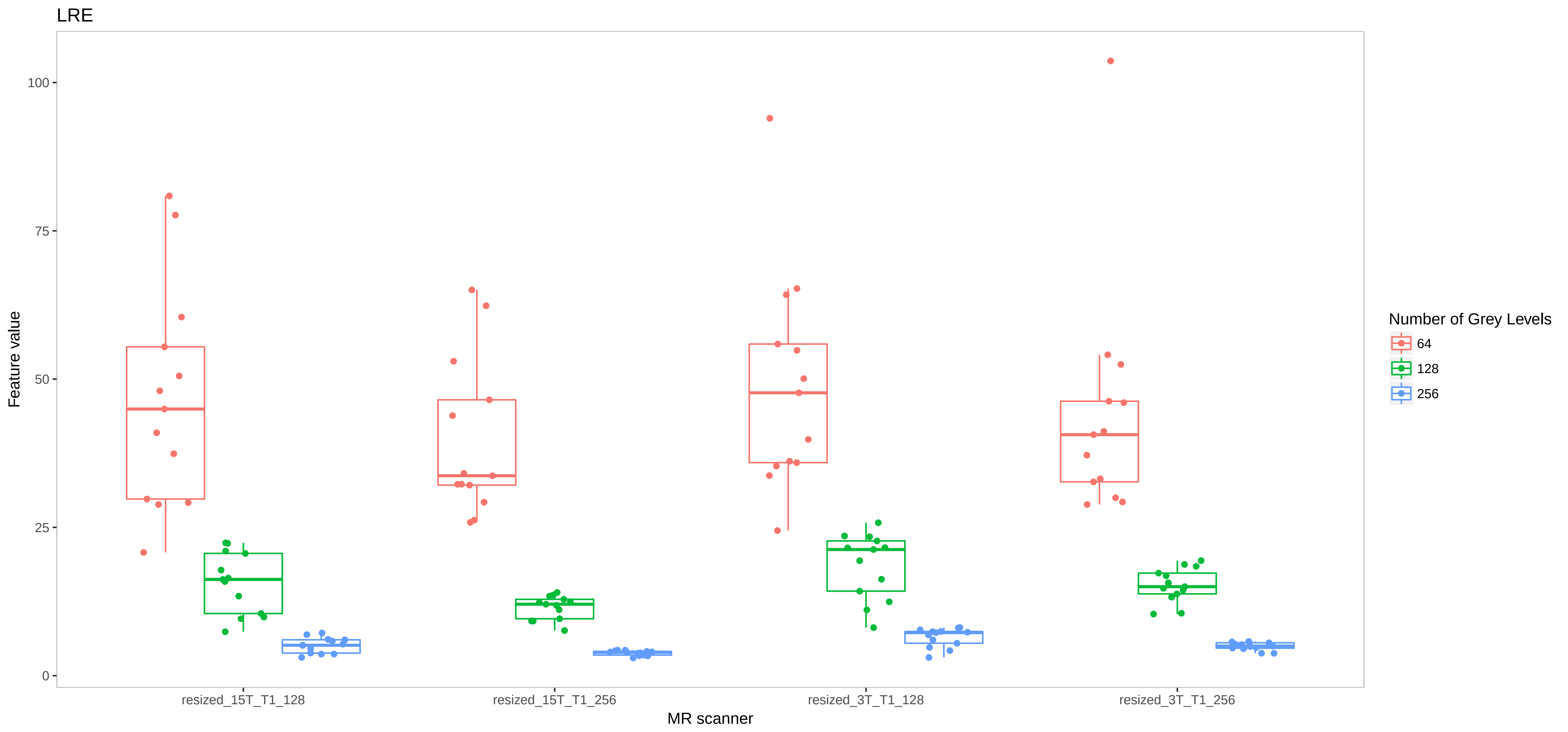
LRE
Feature value
100
75
50
25
0
resized_15T_T1_128
resized_15T_T1_256
resized_3T_T1_128
resized_3T_T1_256
MR scanner
Number of Grey Levels
64
128
256

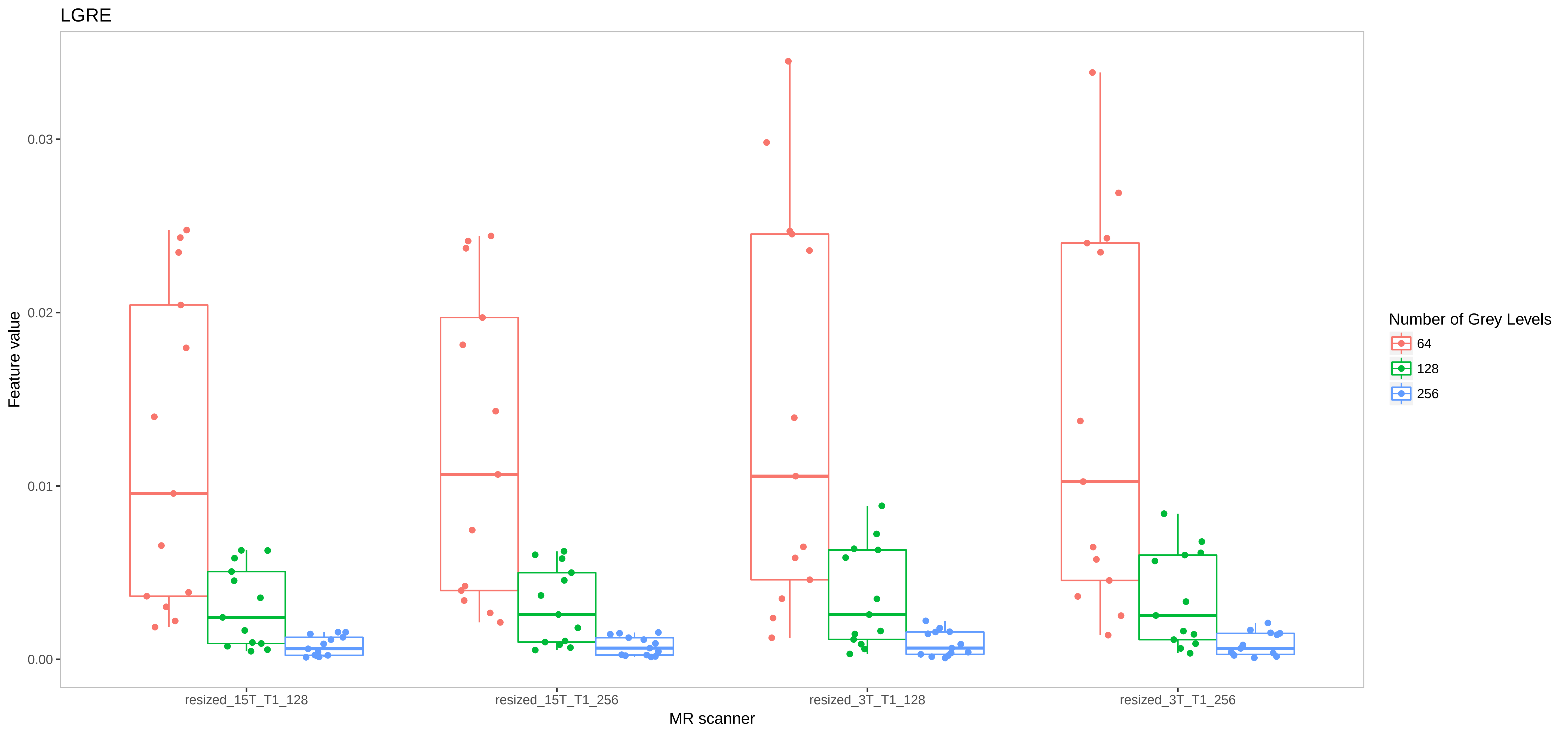
LGRE
Feature value
0.00
0.01
0.02
0.03
resized_15T_T1_128
resized_15T_T1_256
resized_3T_T1_128
resized_3T_T1_256
MR scanner
Number of Grey Levels
64
128
256

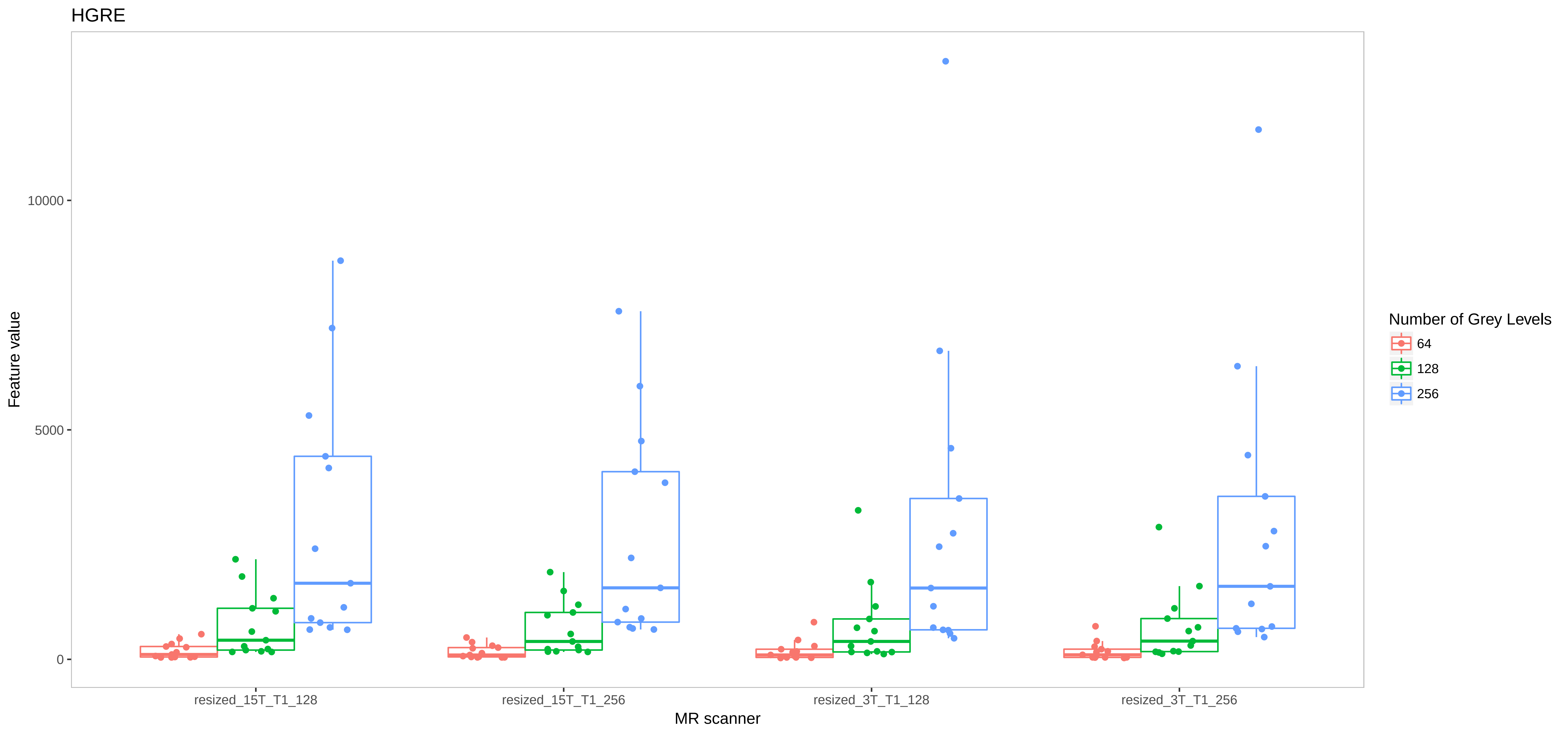
HGRE
Feature value
10000
5000
0
resized_15T_T1_128
resized_15T_T1_256
resized_3T_T1_128
resized_3T_T1_256
MR scanner
Number of Grey Levels
64
128
256

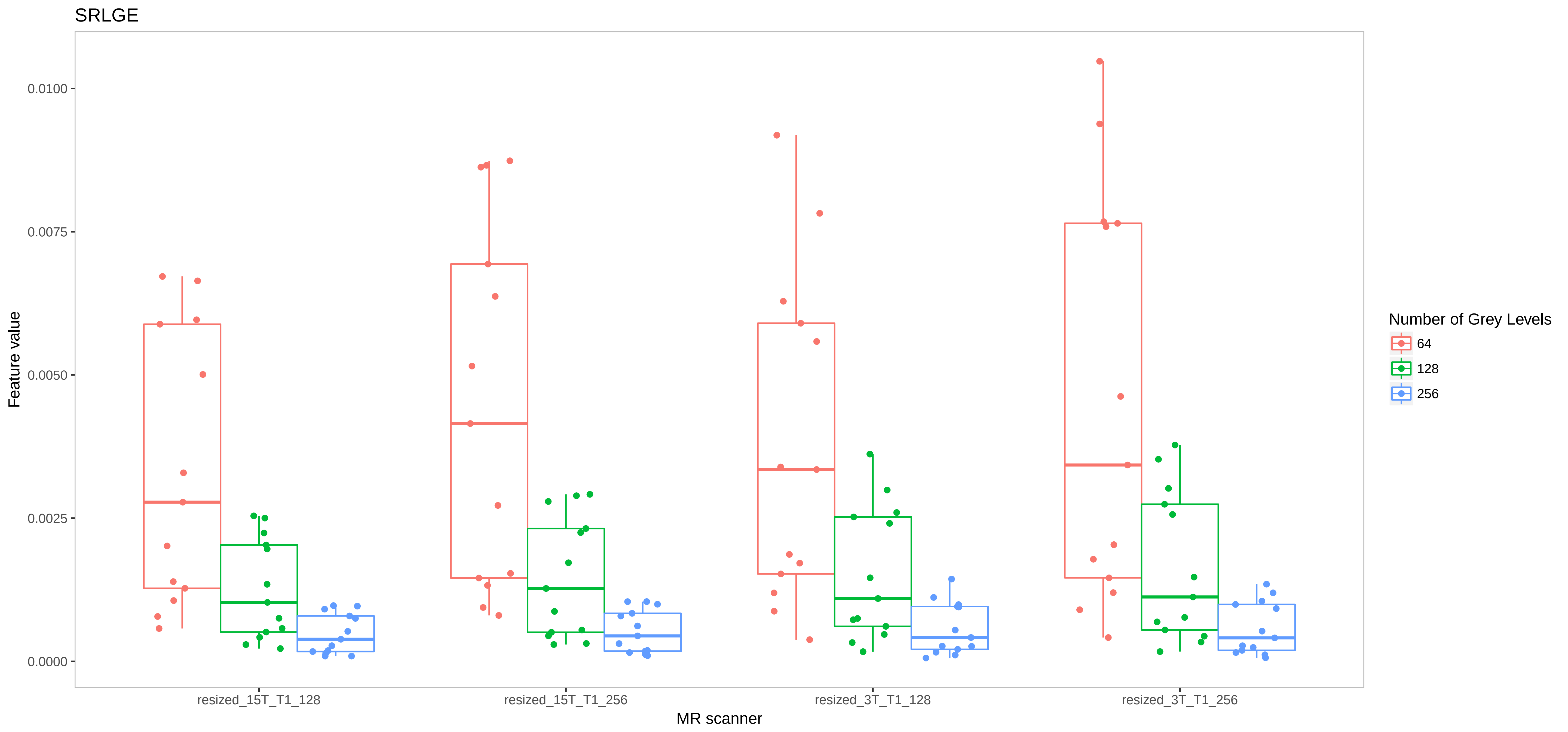
SRLGE
Feature value
0.0100
0.0075
0.0050
0.0025
0.0000
resized_15T_T1_128
resized_15T_T1_256
resized_3T_T1_128
resized_3T_T1_256
MR scanner
Number of Grey Levels
64
128
256

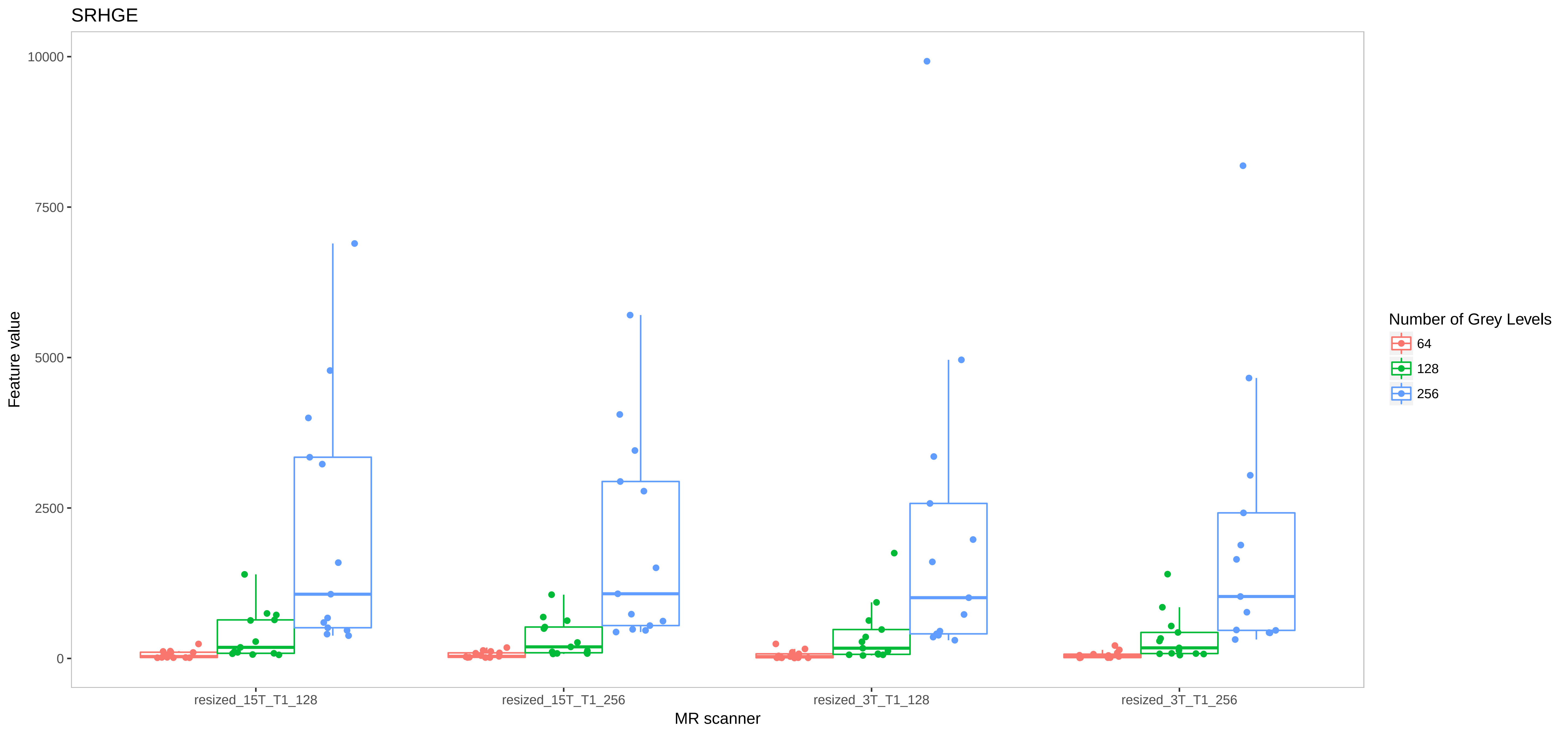
SRHGE
Feature value
10000
7500
5000
2500
0
resized_15T_T1_128
resized_15T_T1_256
resized_3T_T1_128
resized_3T_T1_256
MR scanner
Number of Grey Levels
64
128
256

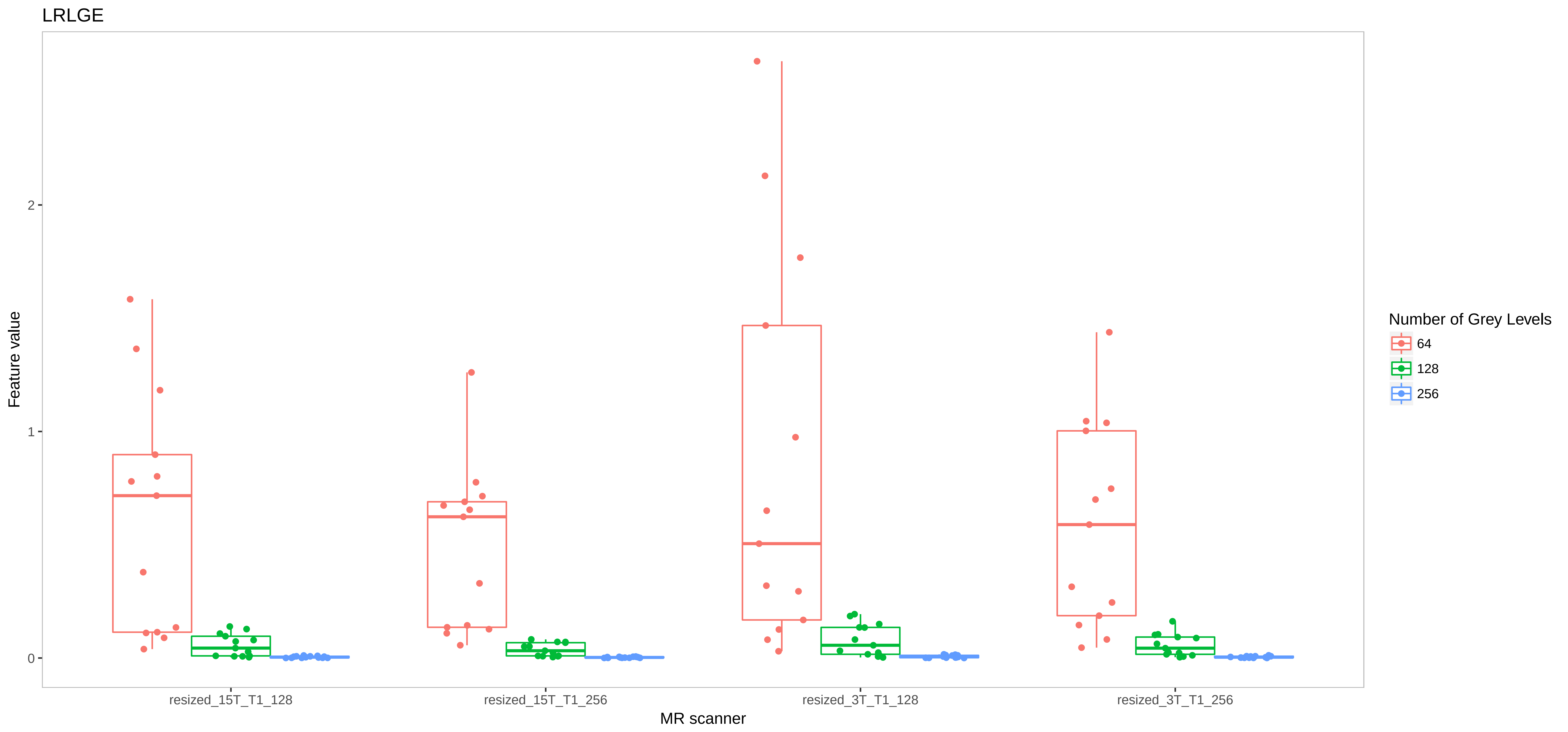
LRLGE
Feature value
2
1
0
resized_15T_T1_128
resized_15T_T1_256
resized_3T_T1_128
resized_3T_T1_256
MR scanner
Number of Grey Levels
64
128
256

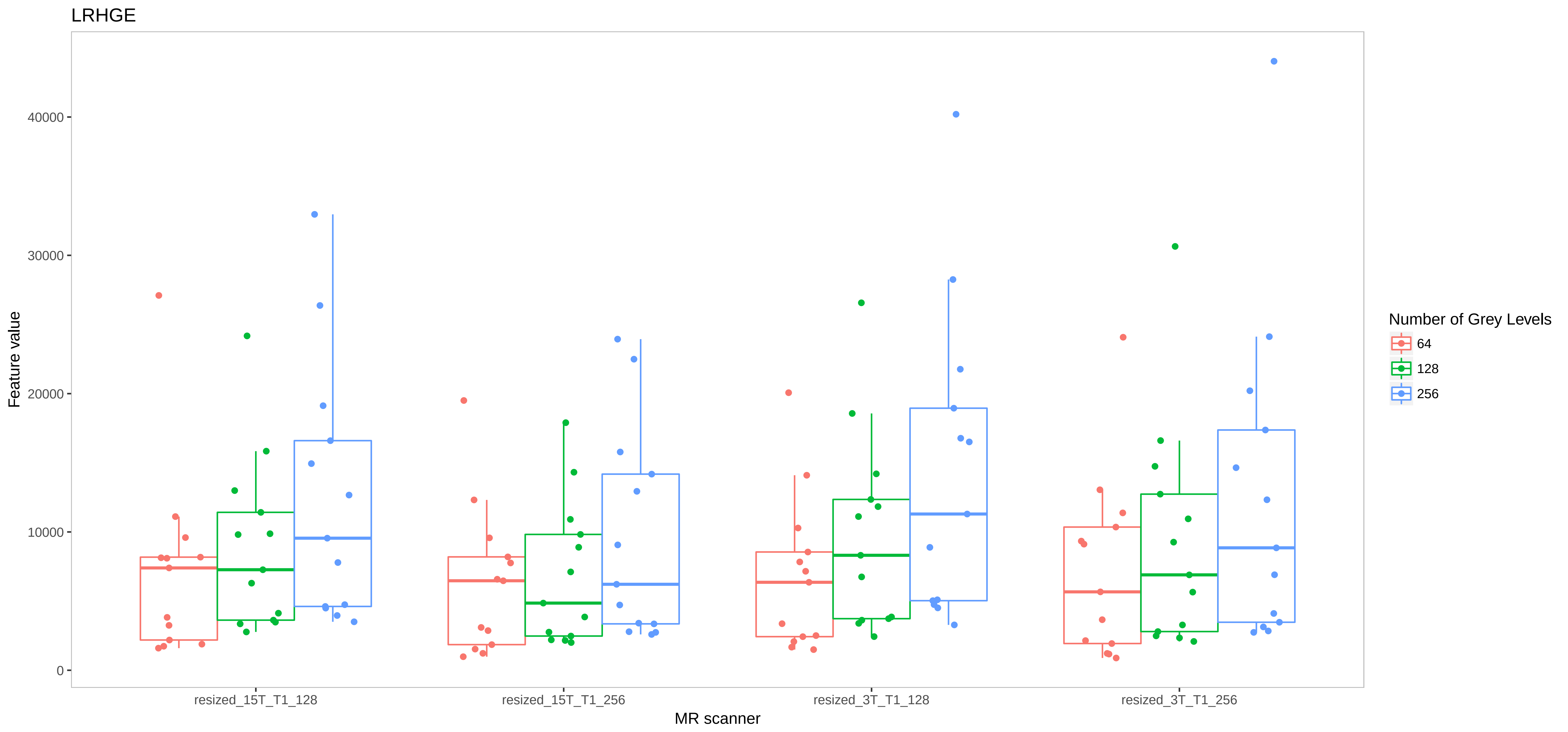

LRHGE
Feature value
40000
30000
20000
10000
0
resized_15T_T1_128
resized_15T_T1_256
resized_3T_T1_128
resized_3T_T1_256
MR scanner
Number of Grey Levels
64
128
256

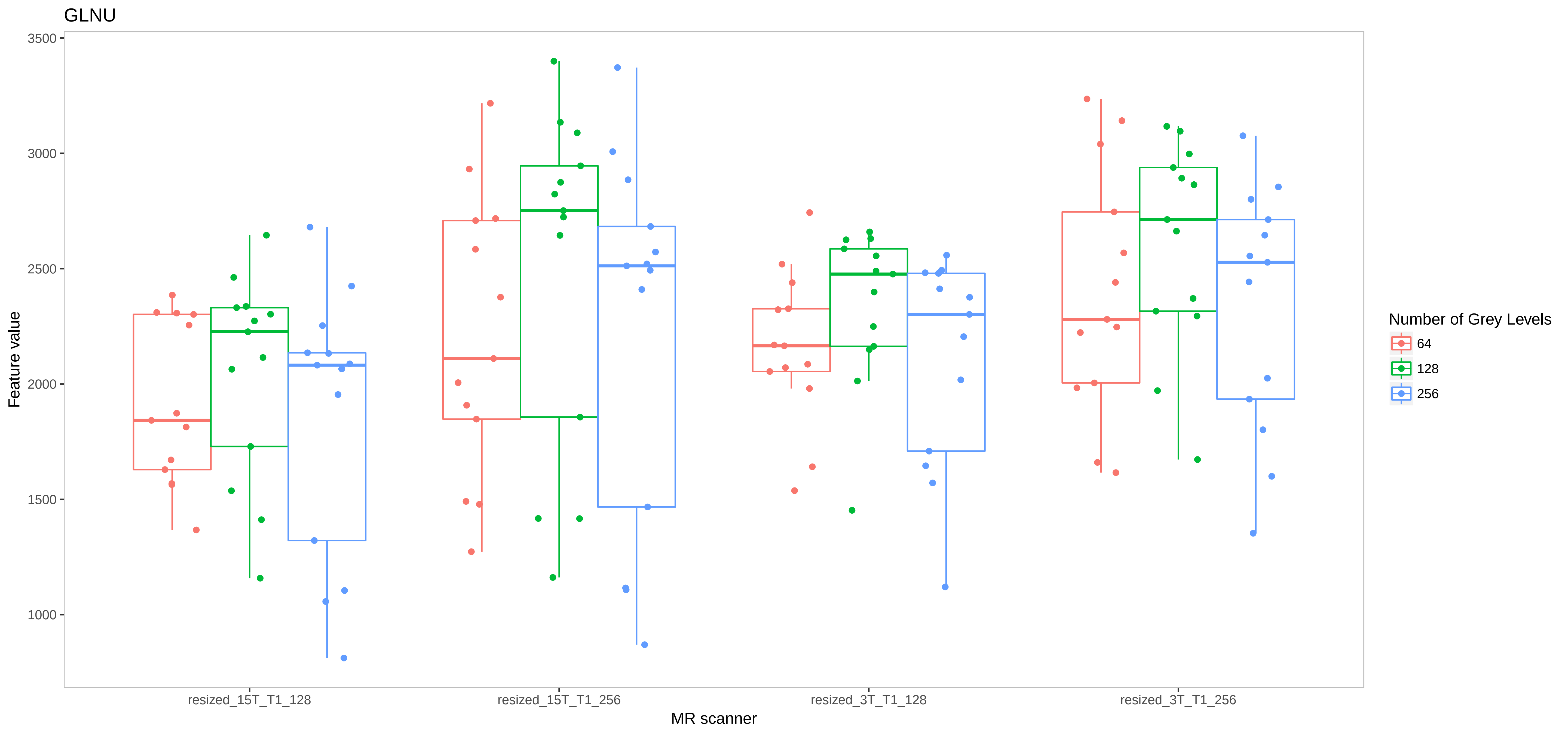
GLNU
Feature value
3500
3000
2500
2000
1500
1000
resized_15T_T1_128
resized_15T_T1_256
resized_3T_T1_128
resized_3T_T1_256
MR scanner
Number of Grey Levels
64
128
256

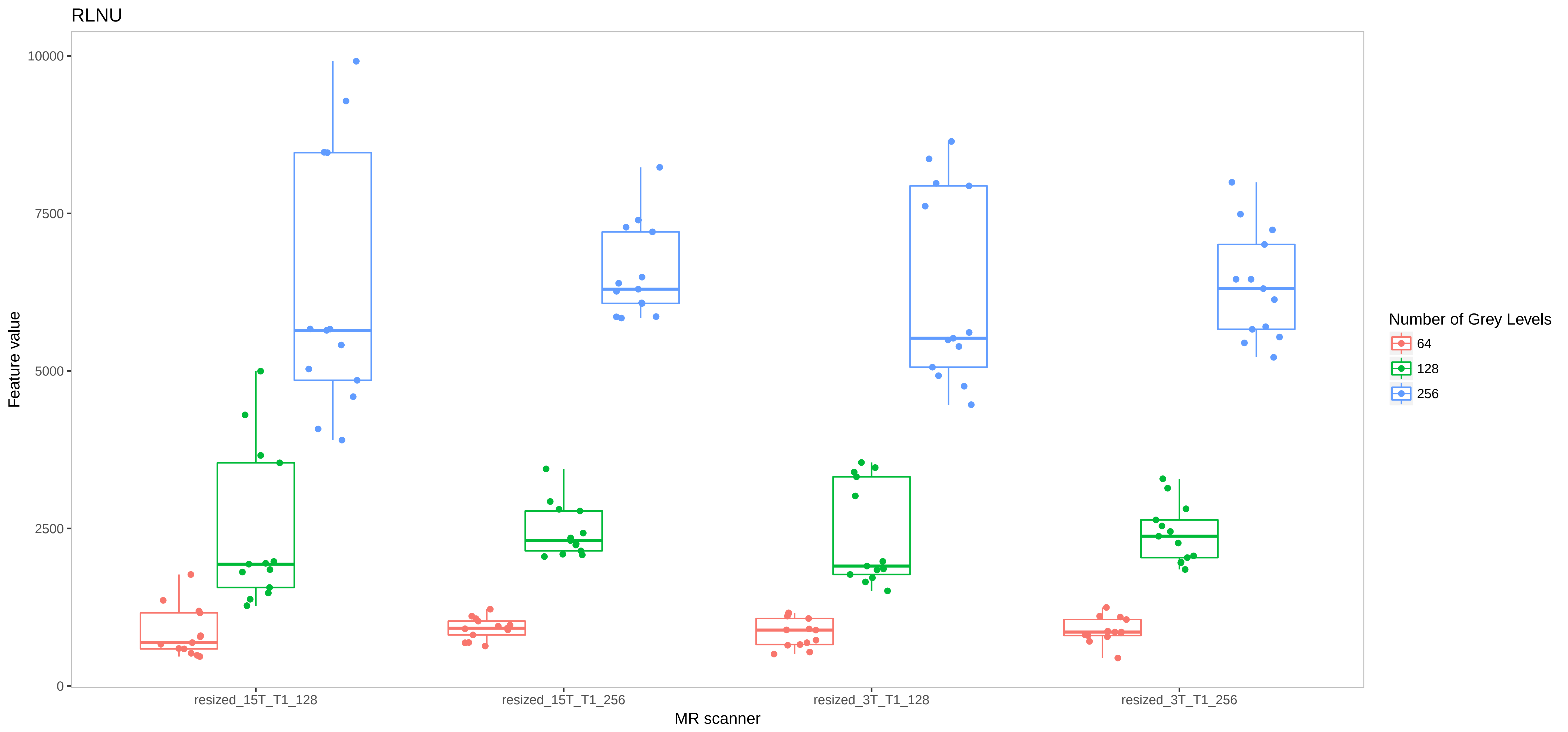
RLNU
10000
7500
5000
2500
0
Feature value
resized_15T_T1_128
resized_15T_T1_256
resized_3T_T1_128
resized_3T_T1_256
MR scanner
Number of Grey Levels
64
128
256

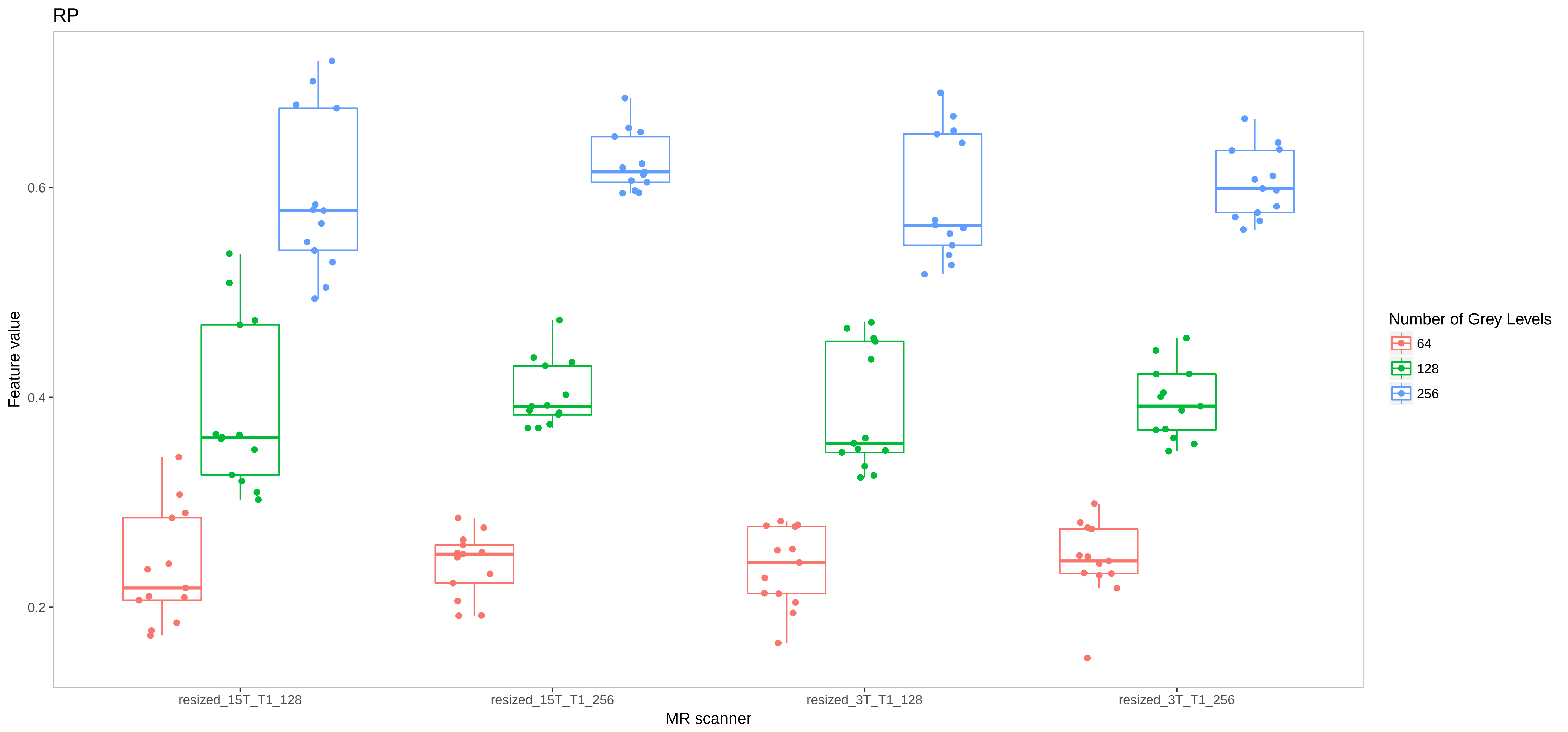
RP
Feature value
0.6
0.4
0.2
resized_15T_T1_128
resized_15T_T1_256
resized_3T_T1_128
resized_3T_T1_256
MR scanner
Number of Grey Levels
64
128
256

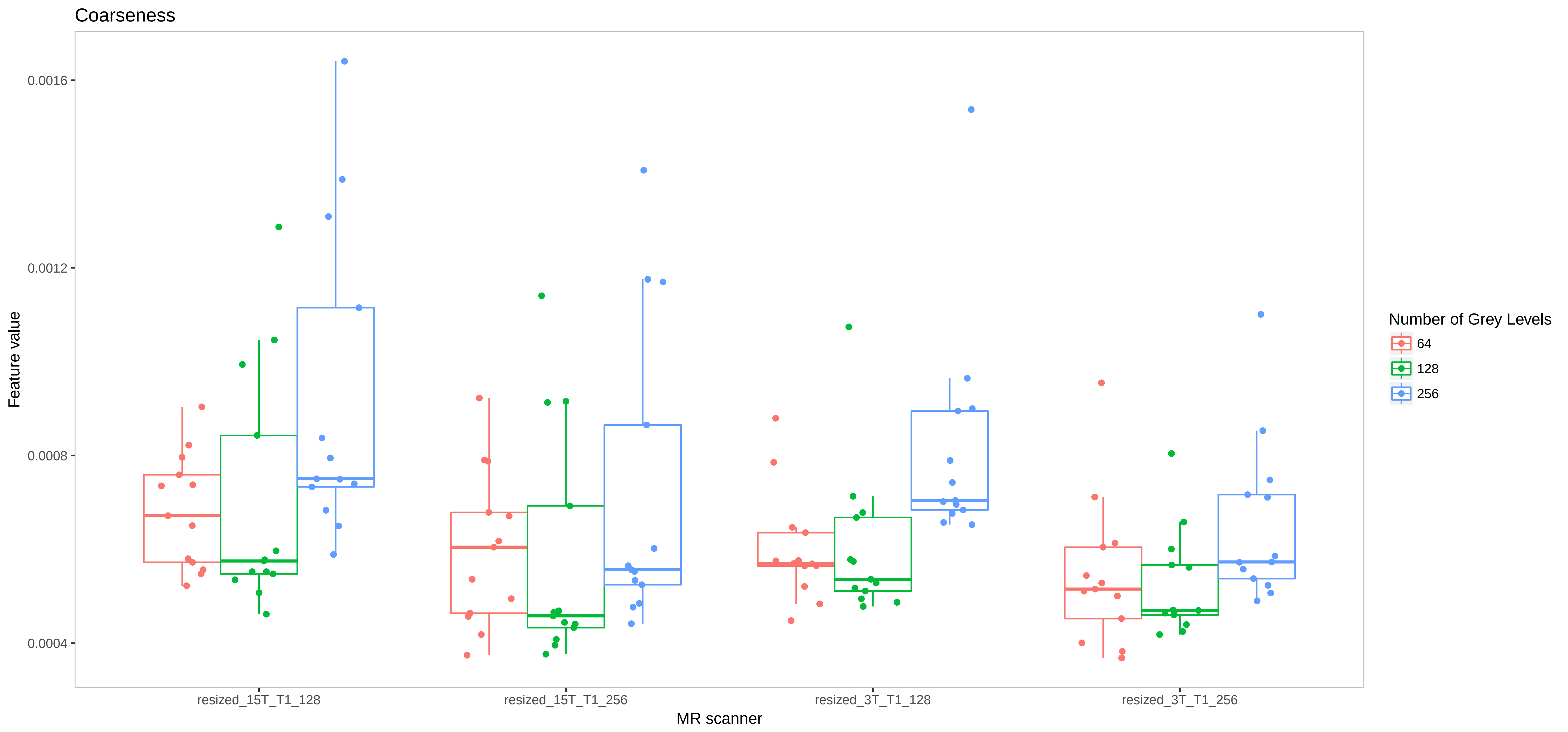
Coarseness
Feature value
0.0016
0.0012
0.0008
0.0004
resized_15T_T1_128
resized_15T_T1_256
resized_3T_T1_128
resized_3T_T1_256
MR scanner
Number of Grey Levels
64
128
256

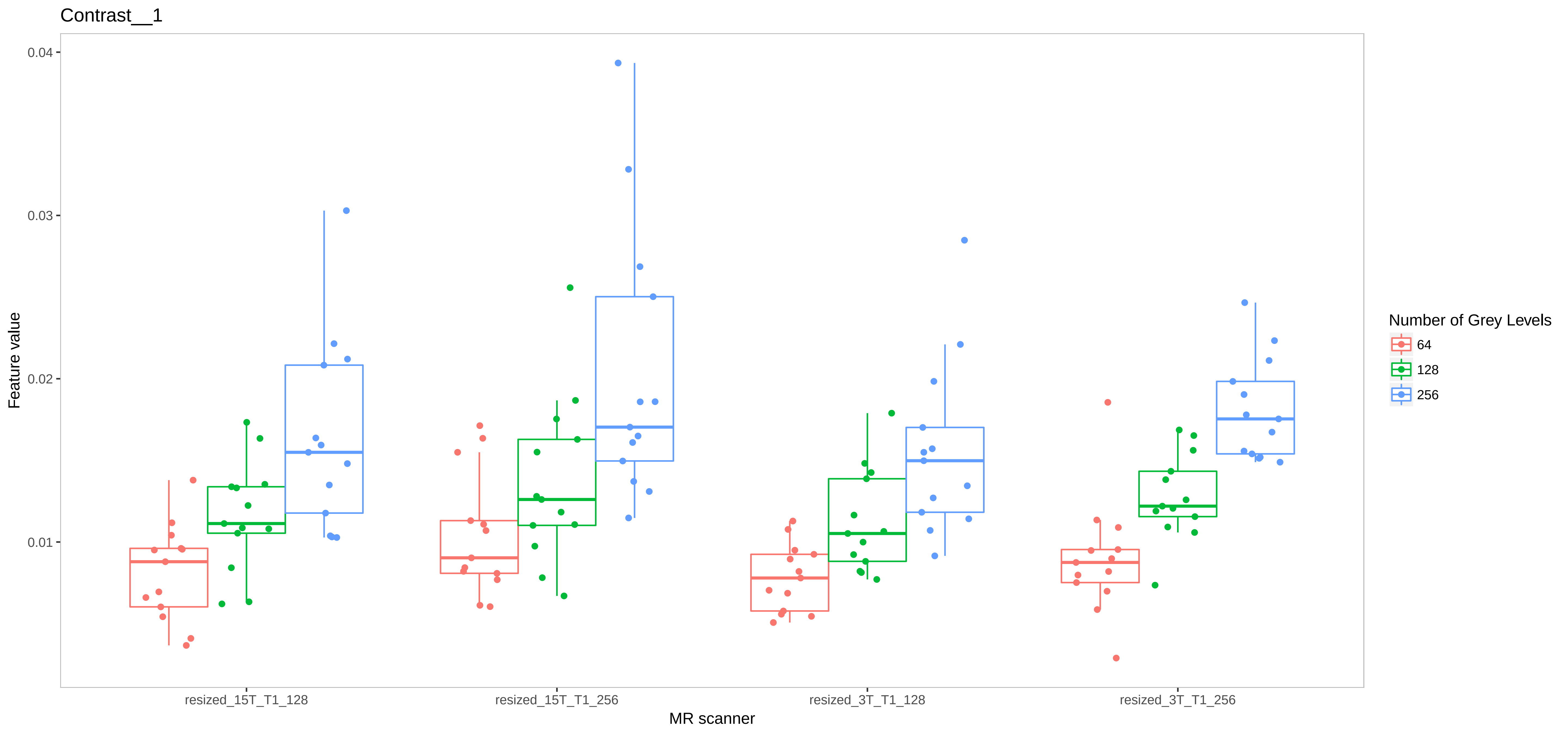
Contrast__1
Feature value
0.04
0.03
0.02
0.01
resized_15T_T1_128
resized_15T_T1_256
resized_3T_T1_128
resized_3T_T1_256
MR scanner
Number of Grey Levels
64
128
256

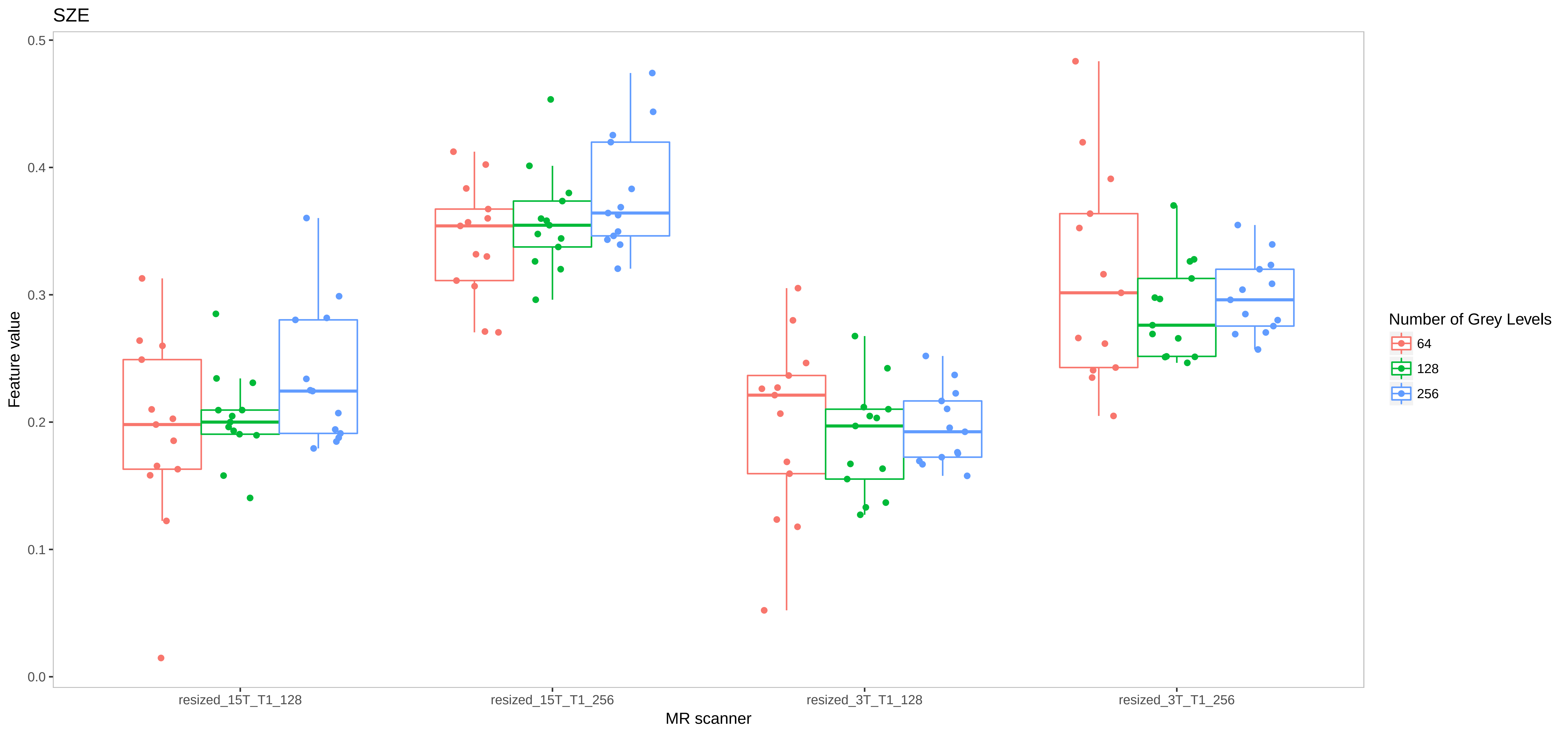

SZE
0.5
0.4
0.3
0.2
0.1
0.0
Feature value
resized_15T_T1_128
resized_15T_T1_256
resized_3T_T1_128
resized_3T_T1_256
MR scanner
Number of Grey Levels
64
128
256

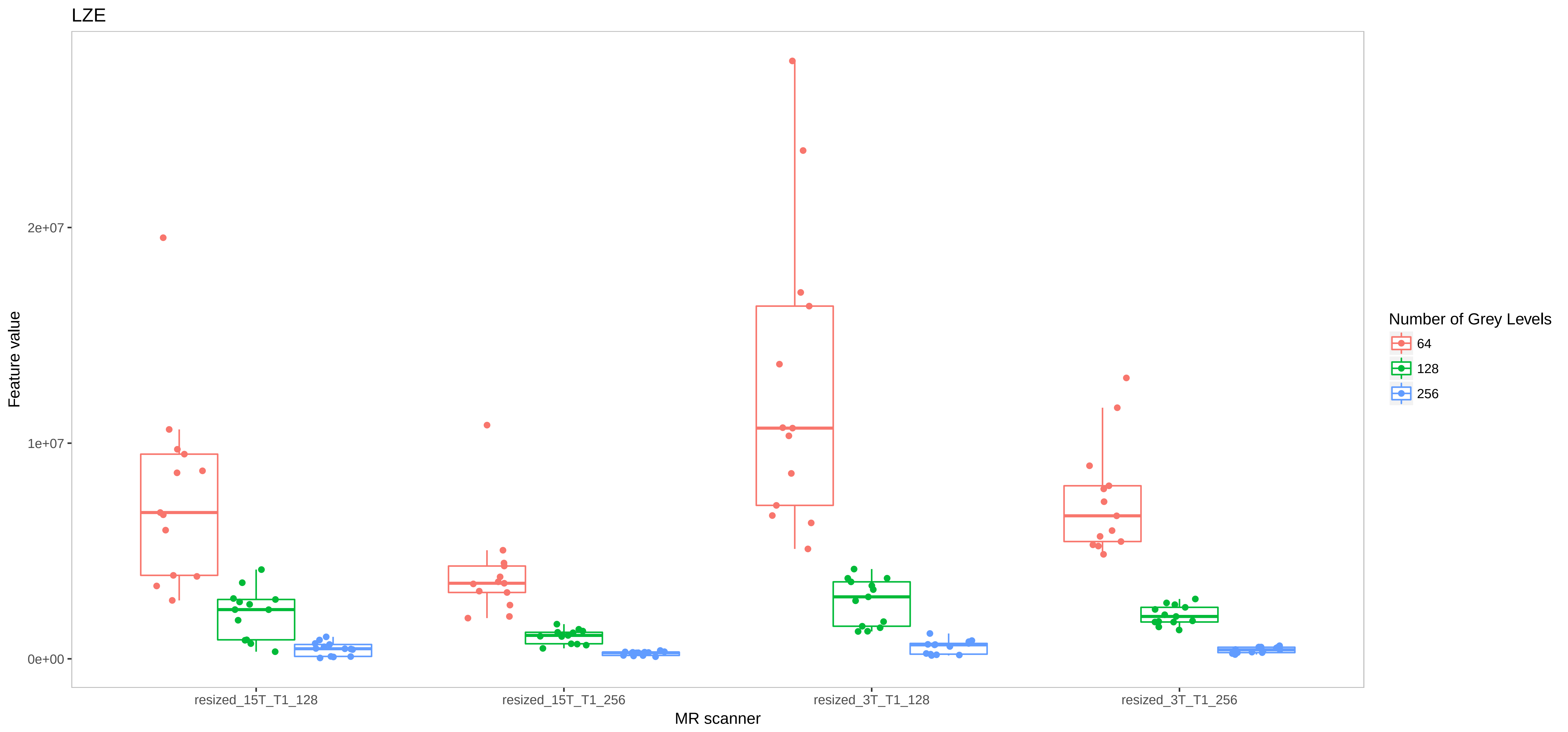
LZE
Feature value
2e+07
1e+07
0e+00
resized_15T_T1_128
resized_15T_T1_256
resized_3T_T1_128
resized_3T_T1_256
MR scanner
Number of Grey Levels
64
128
256

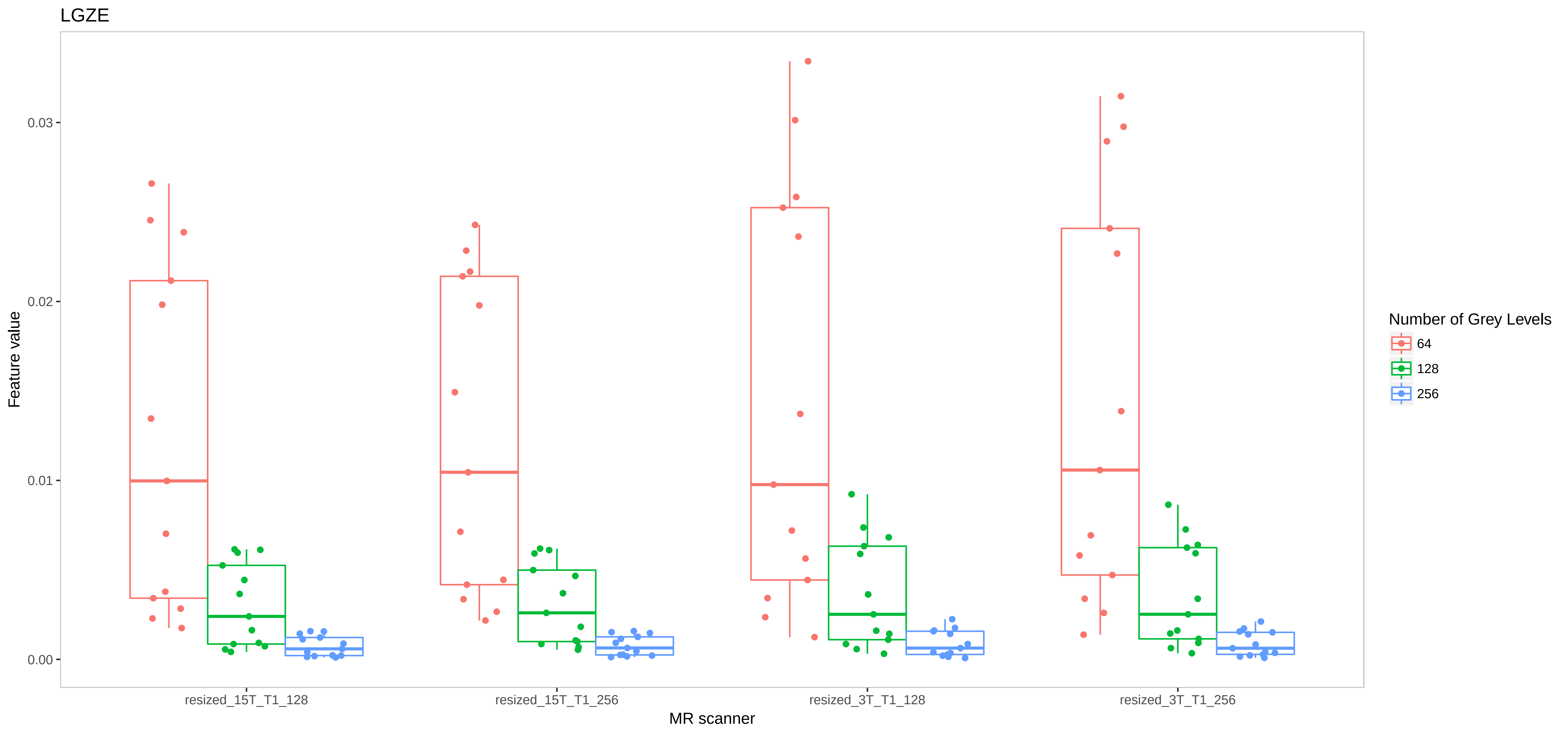
LGZE
Feature value
0.03
0.02
0.01
0.00
resized_15T_T1_128
resized_15T_T1_256
resized_3T_T1_128
resized_3T_T1_256
MR scanner
Number of Grey Levels
64
128
256

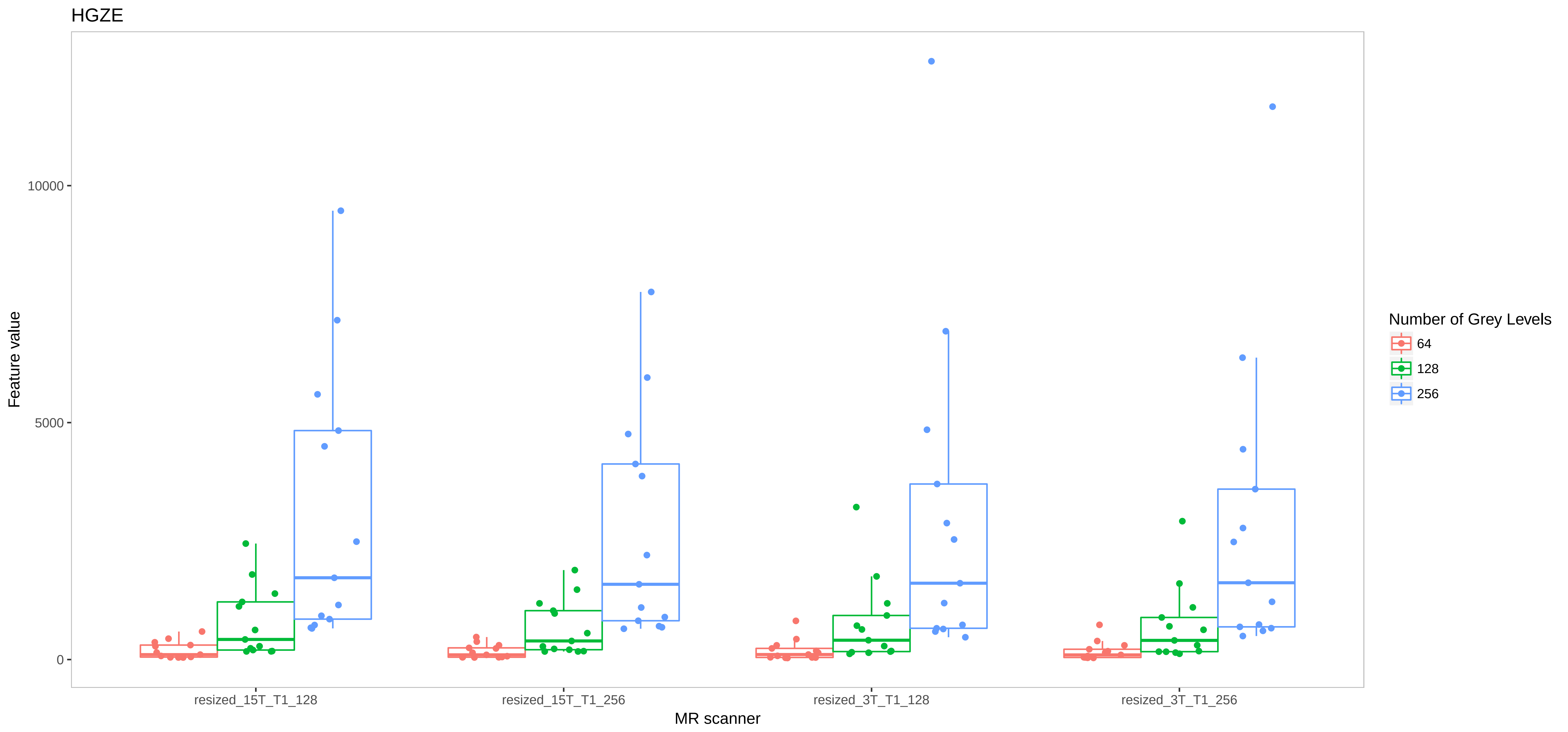
HGZE
Feature value
10000
5000
0
resized_15T_T1_128
resized_15T_T1_256
resized_3T_T1_128
resized_3T_T1_256
MR scanner
Number of Grey Levels
64
128
256

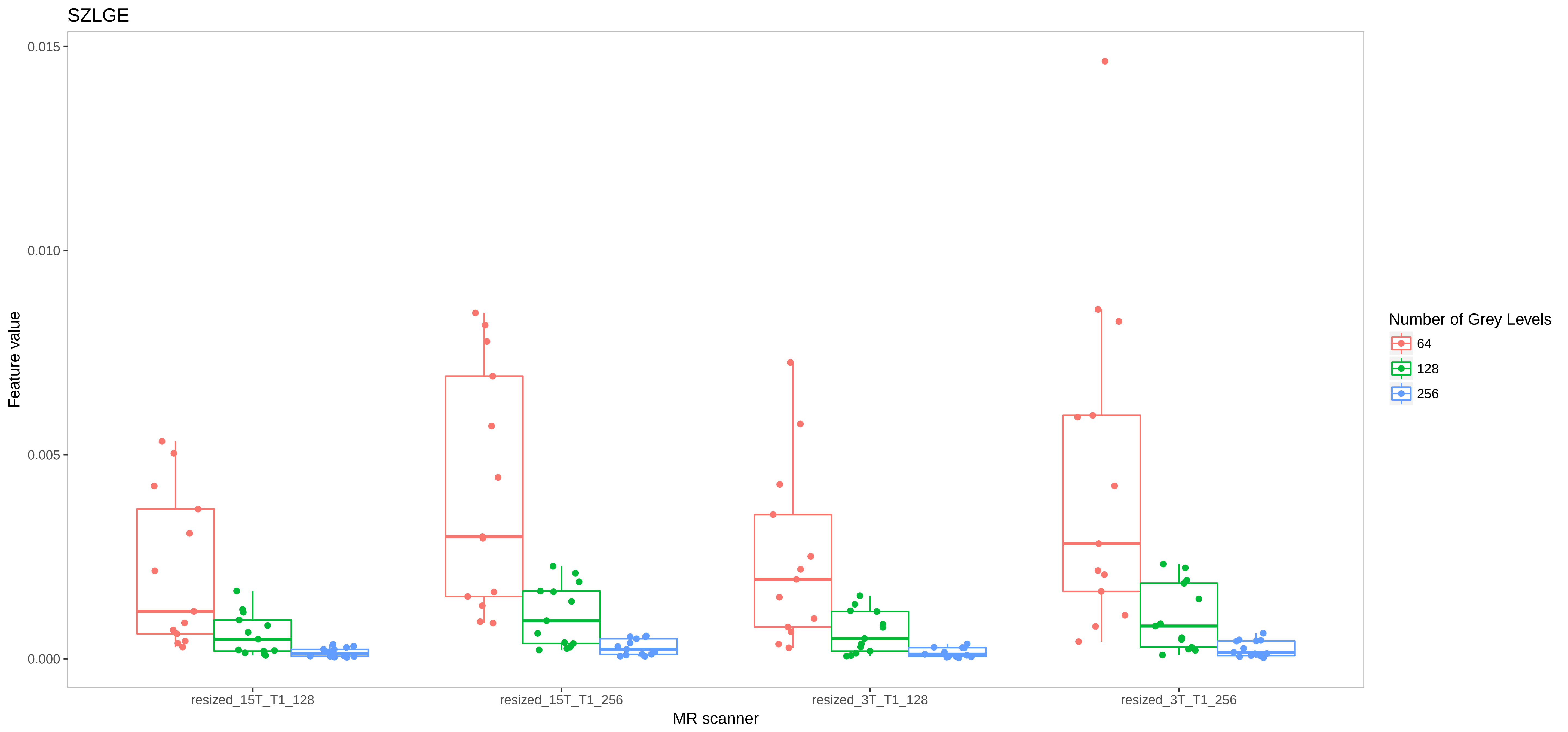
SZLGE
0.015
0.010
0.005
0.000
Feature value
resized_15T_T1_128
resized_15T_T1_256
resized_3T_T1_128
resized_3T_T1_256
MR scanner
Number of Grey Levels
64
128
256

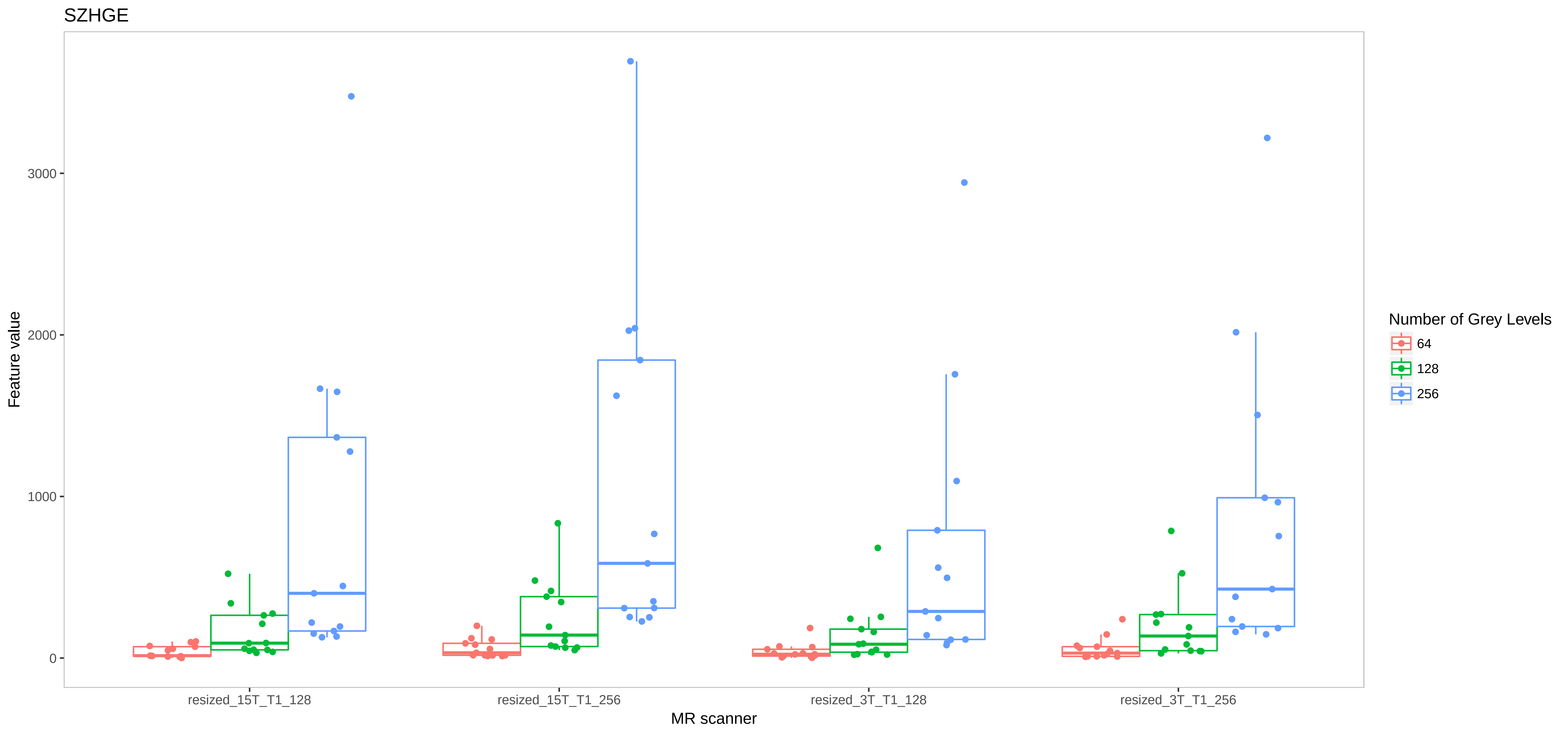

SZHGE
Feature value
3000
2000
1000
0
resized_15T_T1_128
resized_15T_T1_256
resized_3T_T1_128
resized_3T_T1_256
MR scanner
Number of Grey Levels
64
128
256

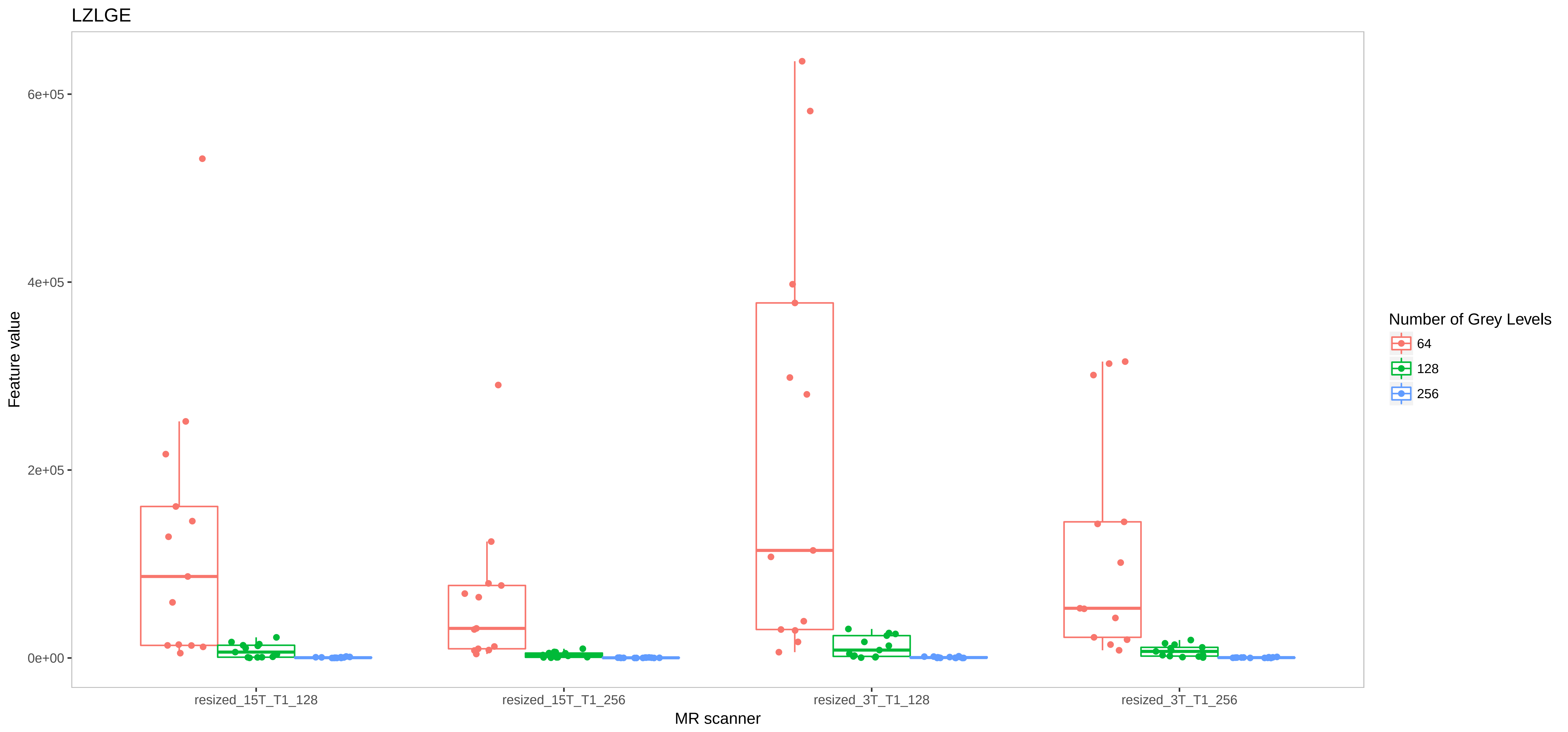
LZLGE
6e+05
4e+05
2e+05
0e+00
Feature value
resized_15T_T1_128
resized_15T_T1_256
resized_3T_T1_128
resized_3T_T1_256
MR scanner
Number of Grey Levels
64
128
256

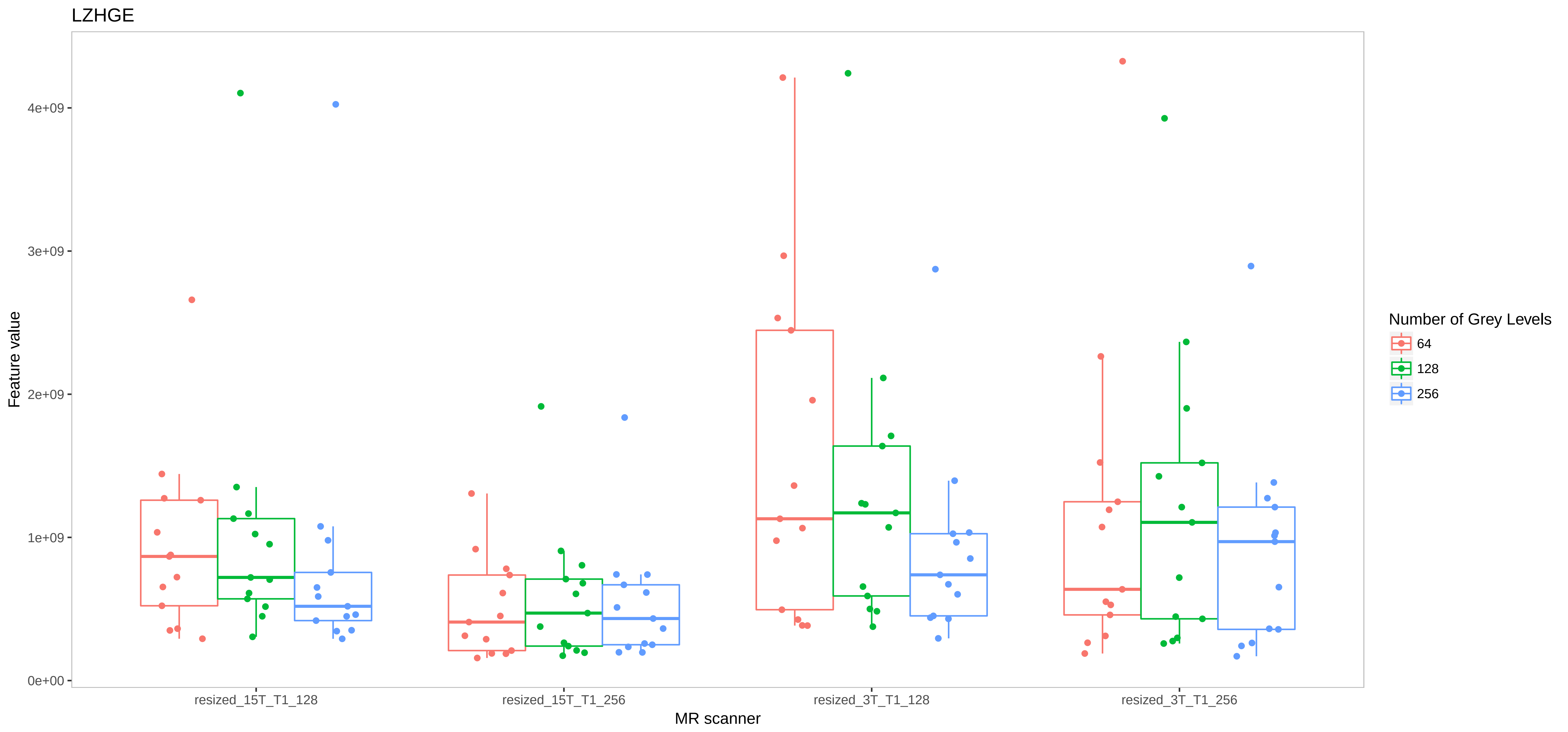
LZHGE
Feature value
4e+09
3e+09
2e+09
1e+09
0e+00
resized_15T_T1_128
resized_15T_T1_256
resized_3T_T1_128
resized_3T_T1_256
MR scanner
Number of Grey Levels
64
128
256

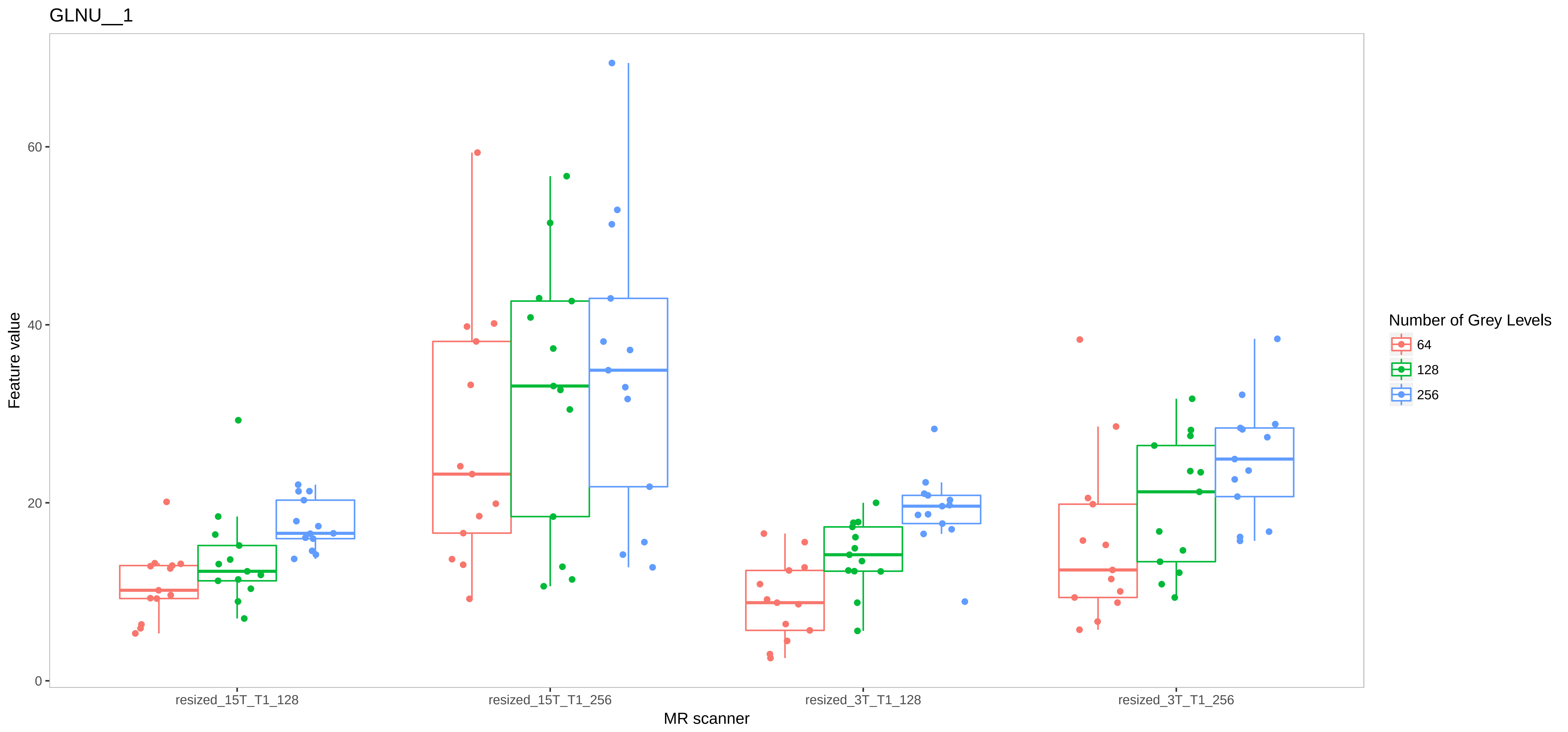

GLNU__1
Feature value
0
20
40
60
resized_15T_T1_128
resized_15T_T1_256
resized_3T_T1_128
resized_3T_T1_256
MR scanner
Number of Grey Levels
64
128
256

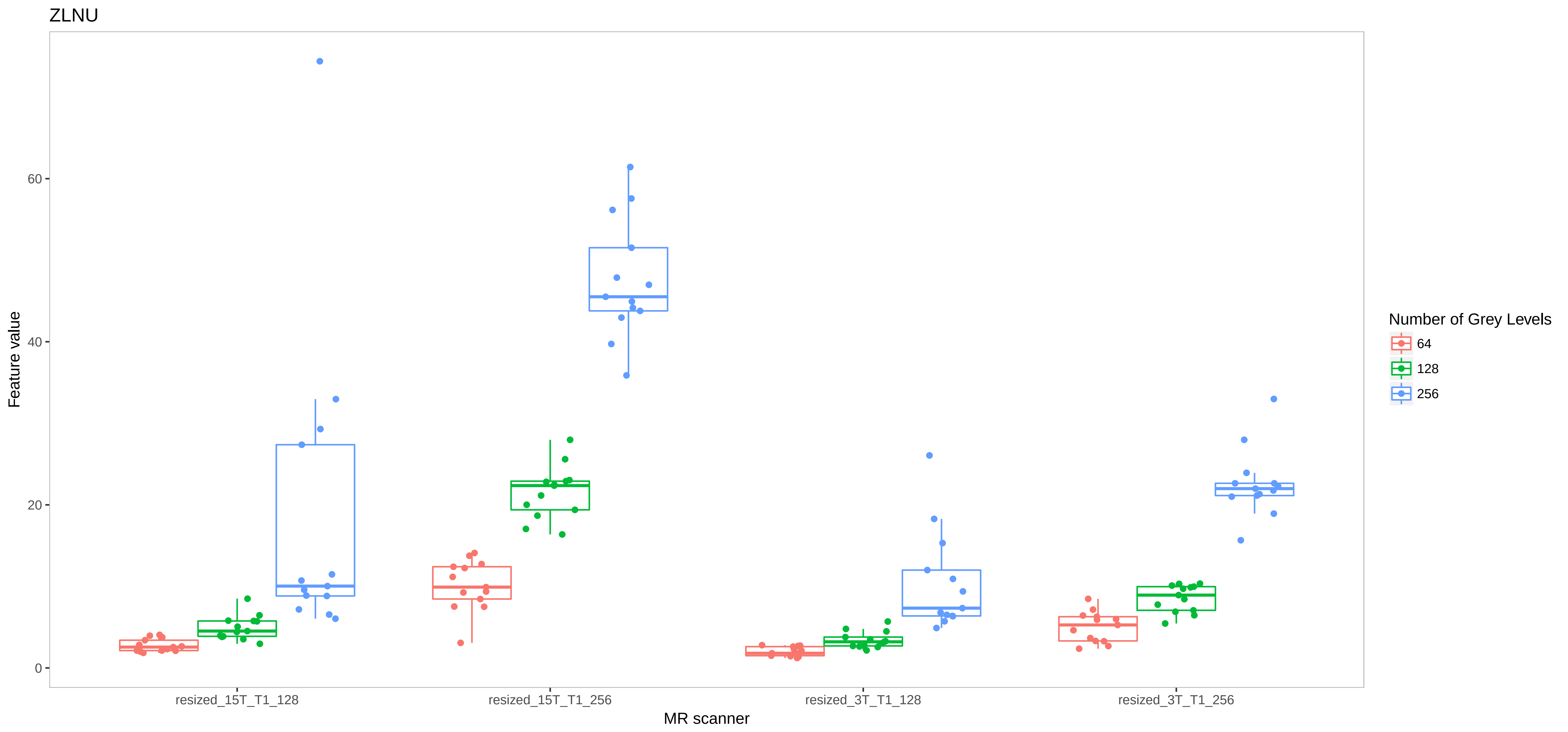
ZLNU
Feature value
0
20
40
60
resized_15T_T1_128
resized_15T_T1_256
resized_3T_T1_128
resized_3T_T1_256
MR scanner
Number of Grey Levels
64
128
256

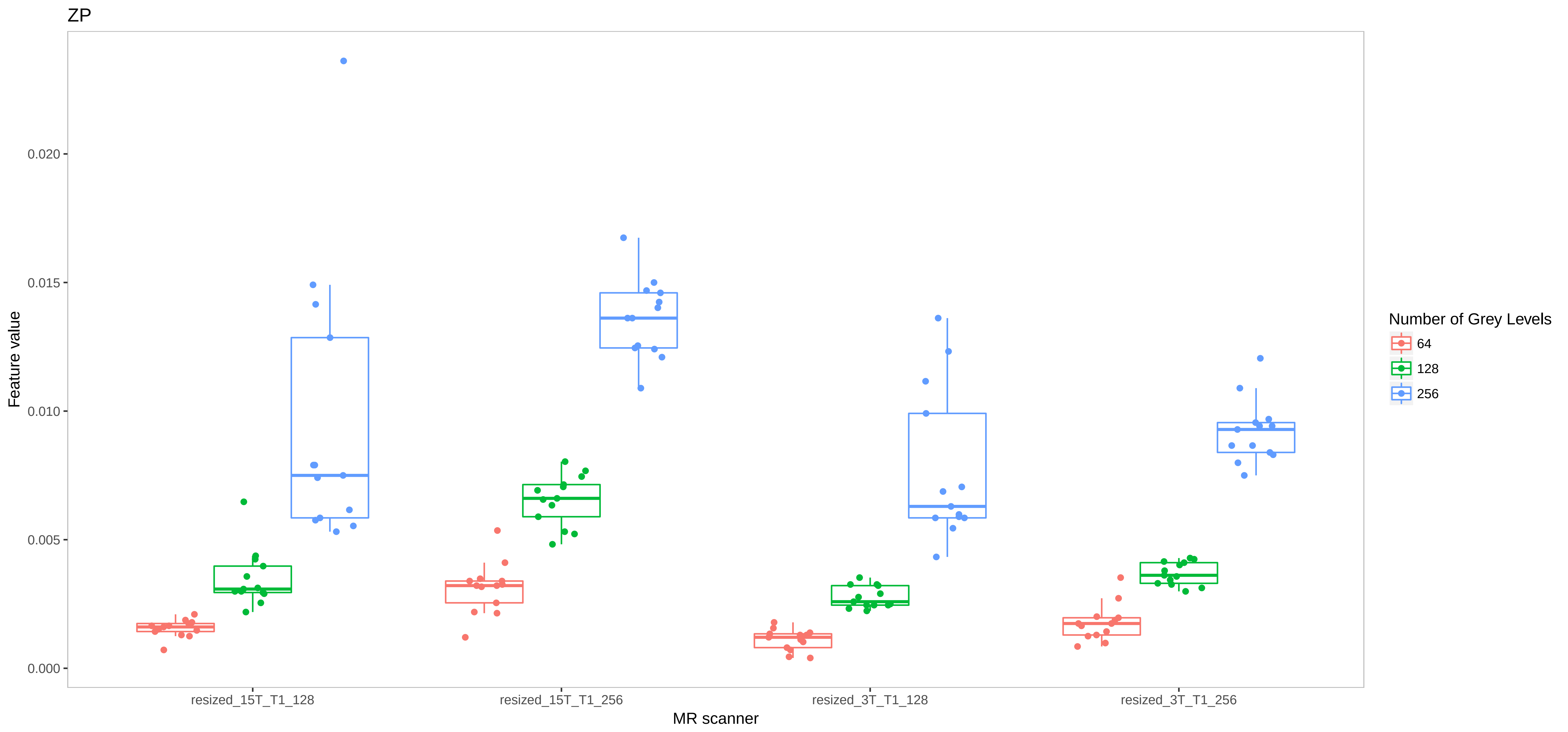

ZP
Feature value
0.020
0.015
0.010
0.005
0.000
resized_15T_T1_128
resized_15T_T1_256
resized_3T_T1_128
resized_3T_T1_256
MR scanner
Number of Grey Levels
64
128
256
