# Supplementary figures and images for: Influence of Magnetic Field Strength on Magnetic Resonance Imaging Radiomics Features in Brain Imaging, an In Vitro and In Vivo Study
Source: Front Oncol. 2021 Jan 20;10:541663. doi: 10.3389/fonc.2020.541663 (PMC7855708; doi:10.3389/fonc.2020.541663)

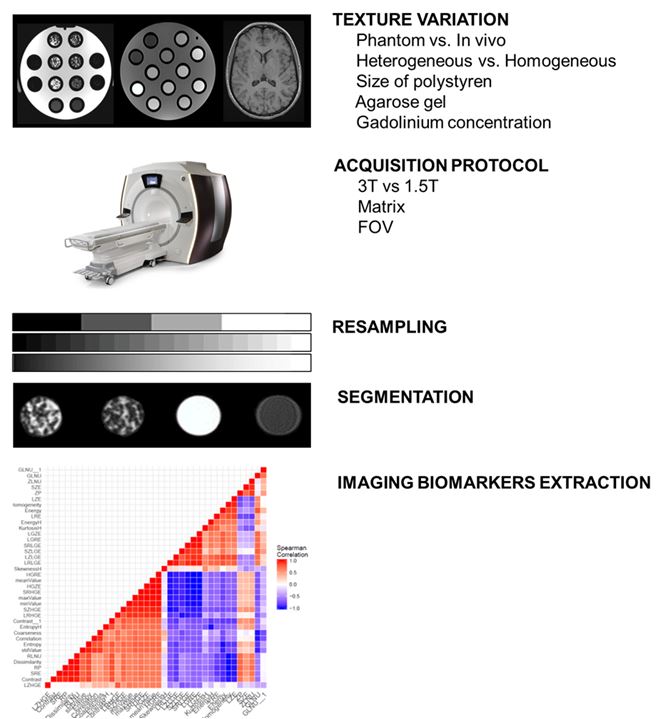

Supplement: Supplementary file 3 [file Image_1.jpeg]

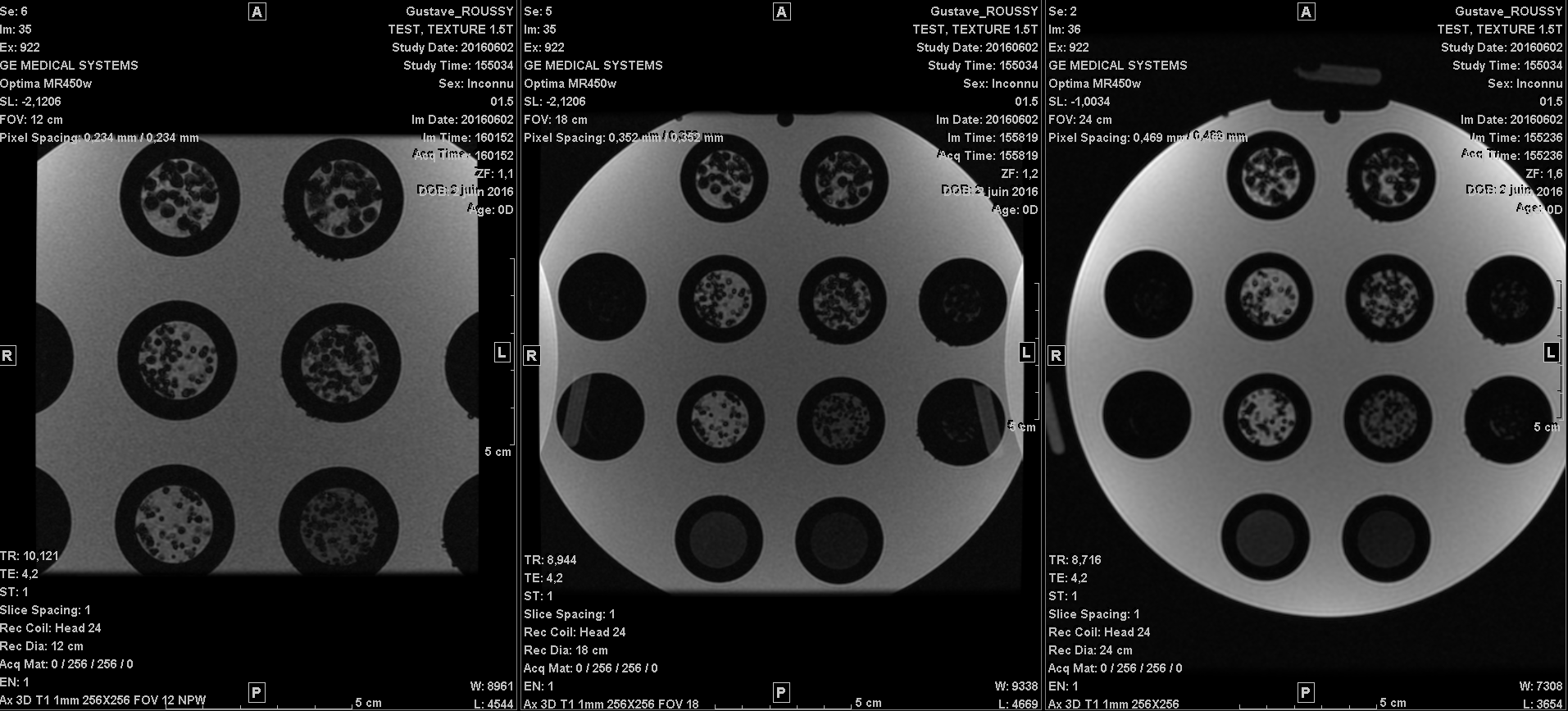

Supplement: Supplementary file 4 [file Image_2.png]
